# Venous Endothelial Marker COUP-TFII Regulates the Distinct Pathologic Potentials of Adult Arteries and Veins

**DOI:** 10.1038/srep16193

**Published:** 2015-11-05

**Authors:** Xiaofeng Cui, Yao Wei Lu, Vivian Lee, Diana Kim, Taylor Dorsey, Qingjie Wang, Young Lee, Peter Vincent, John Schwarz, Guohao Dai

**Affiliations:** 1 School of Chemistry, Chemical Engineering and Life Sciences, Wuhan University of Technology, Wuhan, China 430070; 2 Department of Biomedical Engineering, Rensselaer Polytechnic Institute, Troy, NY 12180, USA; 3 Center for Biotechnology and Interdisciplinary Studies, Rensselaer Polytechnic Institute, Troy, NY 12180, USA; 4 The Center for Cardiovascular Sciences, Albany Medical College, Albany, NY 12208, USA; 5 Neural Stem Cell Institute, Rensselaer, NY 12144, USA

## Abstract

Arteries and veins have very different susceptibility to certain vascular diseases such as atherosclerosis and vascular calcification. The molecular mechanisms of these differences are not fully understood. In this study, we discovered that COUP-TFII, a transcription factor critical for establishing the venous identity during embryonic vascular development, also regulates the pathophysiological functions of adult blood vessels, especially those directly related to vascular diseases. Specifically, we found that suppression of COUP-TFII in venous ECs switched its phenotype toward pro-atherogenic by up-regulating the expression of inflammatory genes and down-regulating anti-thrombotic genes. ECs with COUP-TFII knockdown also readily undergo endothelial-to-mesenchymal transition (EndoMT) and subsequent osteogenic differentiation with dramatically increased osteogenic transcriptional program and calcium deposition. Consistently, over-expression of COUP-TFII led to the completely opposite effects. *In vivo* validation of these pro-atherogenic and osteogenic genes also demonstrates a broad consistent differential expression pattern in mouse aorta *vs.* vena cava ECs, which cannot be explained by the difference in hemodynamic flow. These data reveal phenotypic modulation by different levels of COUP-TFII in arterial and venous ECs, and suggest COUP-TFII may play an important role in the different susceptibilities of arteries and veins to vascular diseases such as atherosclerosis and vascular calcification.

COUP-TFII (Chicken ovalbumin upstream promoter transcription factor 2), also known as NR2F2 (nuclear receptor subfamily 2, group F, member 2), is an orphan member of the steroid receptor superfamily. COUP-TFII is widely expressed in a variety of tissues in the body, and plays an important role in the function and homeostasis of many tissues and organs, such as stem cell differentiation, angiogenesis, lipid/glucose metabolism, and organ development^1^,^2^,^3^,^4^,^5^. Because of its widespread functions, COUP-TFII also has been implicated in pathological conditions such as developmental defects, tumor growth and metastasis^6^,^7^,^8^.

In vascular endothelial cells (ECs), COUP-TFII is expressed at much higher level in venous than arterial endothelium, thus serves as a robust venous EC marker^9^. Previously, venous cell fate is thought to be the default pathway of vascular development as a result of lacking Notch activation. The landmark discovery of COUP-TFII’s function in vein development demonstrated that it is essential for venous specification via suppressing Notch signaling, suggesting that venous pathway is not a default pathway but under active control of COUP-TFII transcription factor^9^. Since then, there have been extensive studies on the molecular mechanisms of how COUP-TFII regulates arterial venous marker expression during embryonic vascular development in zebrafish and mouse model^10^,^11^. However, little is known about whether these developmental programs lead to the differences in pathophysiologic potential of adult arteries and veins, especially those related to vascular diseases such atherosclerosis and vascular calcification. To investigate whether COUP-TFII has a broader role in regulating adult vascular phenotypes beyond the arterial venous specification, we performed comprehensive studies on the role of COUP-TFII in adult EC gene expression patterns, focusing on gene pathways that are directly relevant to the atherosclerosis disease and vascular calcification process. Using a model of COUP-TFII knockdown and over-expression in adult human ECs, we identified the COUP-TFII target genes by transcriptional profiling and further characterized functional phenotypes by using additional molecular and cell biological approaches. These findings were subsequently validated in adult blood vessels in animal models. Besides regulating the known arterial and venous markers, we found COUP-TFII is a critical factor in determining multiple gene pathways that are preferentially expressed in adult arterial *vs*. venous ECs, including extracellular matrix (ECM), inflammation, lipid/metabolism, growth factors, junction proteins, vasomotor factors and thrombosis. Specifically, we found that suppression of COUP-TFII in venous ECs switched its phenotype toward pro-atherogenic by up-regulating the expression of inflammatory genes and down-regulating anti-thrombotic genes. We also discovered that COUP-TFII is a strong regulator of the TGFβ/BMP pathway. ECs with COUP-TFII knockdown readily undergo endothelial-to-mesenchymal transition (EndMT) and subsequent osteogenic differentiation with dramatically increased osteogenic transcriptional program and calcium deposition. Consistently, over-expression of COUP-TFII suppressed all these effects, leading to an anti-atherogenic phenotype and less calcium deposition. These data suggest COUP-TFII may play multifaceted roles in defining the distinctive susceptibilities of adult arteries and veins to vascular diseases such as atherosclerosis and vascular calcification.

## Methods

### Human Endothelial Cell Culture

Human saphenous vein endothelial cells (HSVECs, VEC TECHNOLOGIES, INC.) were cultured at 37 °C in 5% CO2 in Endothelial Cell Growth Medium-2 (EGM-2, Lonza). The culture media was changed every two days. HSVECs were routinely passaged onto tissue culture flasks and discarded after 8 passages to ensure representation of key endothelial characteristics. For each experiment, EC were plated at an initial density of 50,000 cells/cm^2^ on 0.1% gelatin (Sigma) coated tissue culture plate. After 24 hours, the confluence of each EC monolayer was verified by phase contrast examination. To validate that COUP-TFII regulated gene patterns are also true in other endothelial cell types, human aortic endothelial cells (HAEC, Lonza) were cultured and selected genes were validated using RT-PCR (supporting Figure S4).

### Lentivirus to Over-express COUP-TFII

The human Nr2f2 (COUP-TFII) cDNA was amplified from pCMV6-XL5-Nr2f2 (OriGene) by PCR, then sub-cloned into pCDH-EF1-T2A-GFP lentiviral vector (System Biosciences) between EcoRI and NotI digestion sites. Sequences of the PCR primer pair are: Forward, GGAATTCGCCACCATGGCAATGGTAGTCAGCAC; Reverse, ATTTGCGGCCGCTTGAATTGCCATATACGGCCA. To package the lentivirus, the pCDH-EF1-Nr2f2-T2A-GFP or the pCDH-EF1-T2A-GFP constructs were co-transfected with pCMV-VSVG and pCMV-dvpr into 293FT cells using X-tremeGENE HP transfection reagent (Roche). Supernatant was harvested 2 and 3 days later and further concentrated by ultra-centrifugation. The virus pellet was re-suspended in PBS and aliquoted, then put into −80 C for storage. Lentiviruses were used at 10 MOI for cell transduction, and undergo puromycin (2 μg/ml) selection to establish stable ECs that permanently over-express COUP-TFII.

### Isolate Fresh RNA from Endothelium of Mouse Aorta and Vena Cava and cDNA synthesis

Endothelium RNA from mouse aorta and vena cava was isolated using protocol previously described with minor modification^12^. Briefly, mice were euthanized with intra-peritoneal injection of sodium pentobarbital according to protocol approved by Albany Medical College’s Institutional Animal Care and Use Committee (IACUC). Animal’s vasculature was pressure perfused with saline solution with 10 U/ml heparin for 2 minutes via left ventricle after severing inferior vena cava. Aorta and inferior vena cava were isolated, and peri-adventitial fat were carefully cleaned in ice-cold PBS. The lumen of the vessels was quickly flushed with 300 ul ice-cold TRIzol reagent (Invitrogen) using 25 G needle into a microcentrifuge tube. Total RNA were isolated and purified from the elute using RNeasy Plus Kit (Qiagen), and subsequent cDNA synthesis using Superscript III First-Strand Synthesis system. To verify the purity of the RNA (without smooth muscle cells contamination), gene expression of alpha-smooth muscle actin (α-SMA) was assessed on the RNA sample and compared to the RNA isolated from the media layer of the blood vessel segment. The purify of the RNA was validated to have <1% α-SMA contamination (supporting Figure S5).

### En face Immunofluorescence Microscopy of Mouse Aorta and Vena Cava

*En face* immunofluorescence microscopy of mouse aortic arch was performed as described previously^13^. FVB/NJ mice were purchased from Jackson Laboratory (Bar Harbor, ME) and maintained in accordance with Institutional Review Board approved protocols. To examine cytokine induced adhesion molecule VCAM-1 expression, TNF-a (30 ug/kg body weight, ~1 ug per mouse) was injected via tail vein of the mouse based an established protocol^14^. After 16 hour, mice were anesthetized and perfused with PBS and 2% paraformaldehyde, aorta and vena cava were then harvested. The vessel was then cut open and suspended in PBS followed by permeabilization in 0.1% Triton X-100/PBS. After blocking with 5.5% FBS in 0.1% Triton X-100/PBS, the aorta was incubated with either mouse anti-BMP4 antibody (1:100; Santa Cruz Biotechnology), or rabbit anti-Cx40 antibody (1:100; Alpha Diagnostic International), or FITC conjugated rat anti–mouse VCAM-1 (1:100; BD Pharmingen). Endothelium was co-stained by goat anti-VE-cadherin antibody (1:100; Santa Cruz Biotechnology) in PBS at 4 °C overnight. Secondary antibodies included Alexa Fluo 488 donkey anti-mouse (1:200; Life Technologies), Alexa Fluo 488 donkey anti-rabbit (1:200; Life Technologies) Alexa Flur 594 donkey anti mouse (1:200, Life Technologies) and Alexa Fluo 647 donkey anti-goat (1:200, Life Technologies), Alexa Fluor 488 goat anti–rat antibody (1:200 Life Technologies).

To identify nuclei, DAPI (1:10,000, Life Technologies) was added during the incubation of secondary antibodies. For the aorta, the lesser curvature of the aortic arch and a portion of thoracic aorta were cut and mounted with the endothelium facing up. Images of the *en face* preparation were obtained using a Zeiss LSM META 510 confocal microscope system with Zeiss Zen software, or Zeiss Axio Observer Z1 inverted microscope with the Apotome module and Zeiss AxioVision software.

### Assay the Osteogenic Potential of Cultured ECs

To assay the osteogenic potential of cultured HSVECs, the culture medium was switched from EGM-2 to StemPro osteogenesis differentiation medium (Life Technologies). After 7 days of culture, RNA was isolated and Taqman RT-PCR was performed to assess the gene expression involved in osteogenic pathway: RUNX2, SP7, DLX5, ALPL, COL1A1, IBSP, BGLAP, SPP1 and BMP4. To further validate the functional consequences of the osteogenic potential, Alkaline Phosphatase (ALP) and Alizarin Red S staining were performed on cells following 7 days of culture under osteogenic differentiation medium. Briefly, for Alkaline Phosphatase staining, cells were rinsed twice by PBS and then fixed using 1 mL 10% formaldehyde in each well for 1 min (fixing cells longer than 2 min will result in the inactivation of alkaline phosphatase). After the fixation, the fixative was aspirated and the cells were rinsed again using PBS. ALP activities were detected by SigmaFast BCIP/NBT (Sigma-Aldrich) following the manufacturer protocol. For Alizarin Red S staining, cells were rinsed twice using calcium and magnesium free PBS and then fixed using 1 mL 10% formaldehyde in each well for 10 min. Cells were then washed twice using PBS and stained using an Alizarin Red S kit (American MasterTech) to evaluated the calcium deposit following the manufacturer protocol.

Additional materials and methods are described in detail in Supporting Information.

## Results

### COUP-TFII Regulates Multiple Gene Pathways Related To Atherosclerosis In Adult ECs

**Figure 1:**
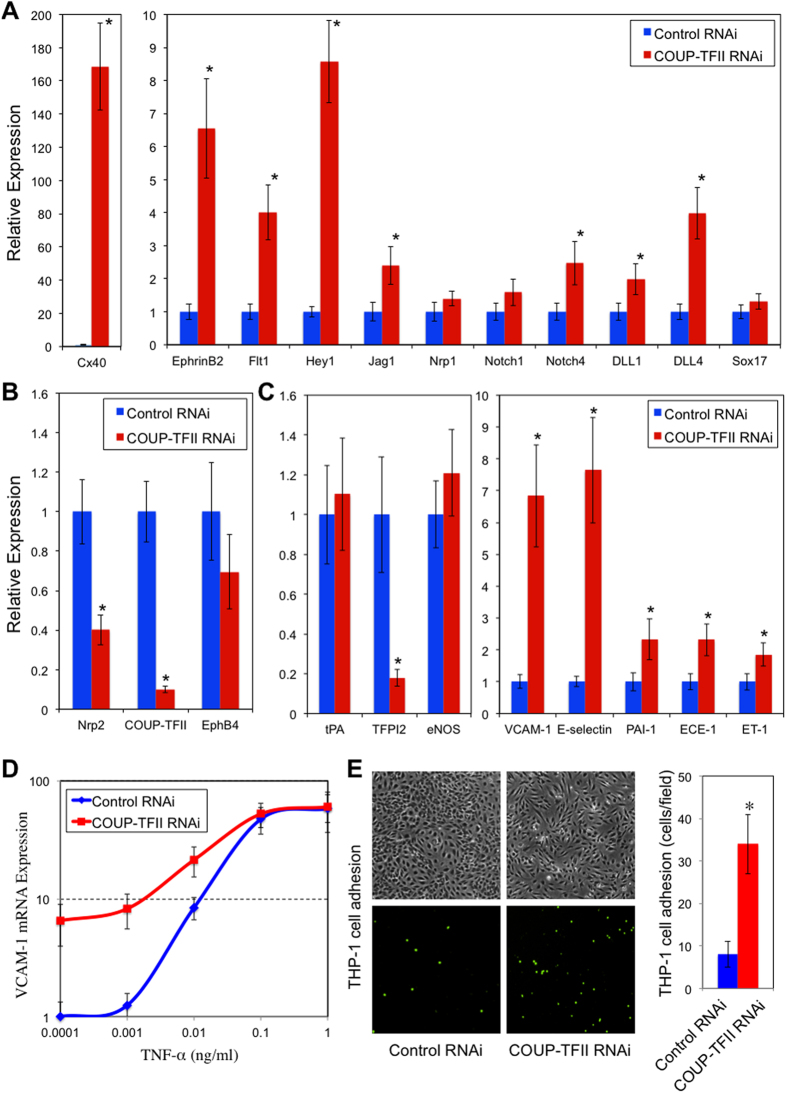
COUP-TFII not only controls molecular markers of arterial and venous identity, but also regulates EC genes of inflammation, thrombosis and vasomotor functions. (**A**–**C**) HSVECs were transfected with COUP-TFII or Control RNAi (10 nM). RNA was collected 72 hours after transfection. Gene expressions were measured by Taqman-RT PCR and normalized to RNA 18S. Data are represented as relative expression level, n = 3, *p < 0.05. (**D**) HSVECs were transfected with COUP-TFII or Control RNAi (10 nM) for 48 hours, and then treated with different doses of TNF-α (0–1 ng/ml) for 24 hours. VCAM-1 gene expressions were measured by Taqman-RT PCR and normalized to RNA 18S, n = 3. (**E**) HSVECs were treated with 0.01 ng/ml TNF-α for 24 hours, and THP-1 cells (labeled with CellTracker Green) adhesion to the EC surface was quantified by counting number of attached cells within each imaging field and averaged over 10 imaging fields. *p < 0.05.

We first applied RNAi to knockdown COUP-TFII expression in cultured human saphenous vein endothelial cells (HSVEC), and performed transcriptional profiling of the genes that are differentially expressed. As shown in Table S1 (in Supporting Information), knockdown of COUP-TFII leads to the increase of selected arterial markers such as Ephrin-B2, Hey1,2, Dll1,4, Flt1, Notch4, Jag1, Hes4, Foxc2, which is consistent with the role of COUP-TFII in venous specification as reported before^9^. The regulation pattern of arterial markers (Ephrin-B2, Hey2, DLL4) by COUP-TFII in cultured adult ECs also confirmed the recent study, in which COUP-TFII negatively regulates the Ephrin-B2, Hey2 and DLL4 expression^15^ in human umbilical artery endothelial cells. Several genes involved in angiogenesis/vasculogenesis are also up-regulated including EPAS1/HIF-2a, Ephrin-A1, Tie-2, EphA4. The up-regulated genes also include several previously unknown targets including Sema7a (Semaphorin 7A), a molecule involved in the axon guidance, and Vash1 (Vasohibin 1), an angiogenesis inhibitor. Gap junction proteins (Connexin 37 & 40) are also strongly up-regulated. Meanwhile, knockdown of COUP-TFII reduces the Nrp2, a venous EC marker. We validated several selected arterial and venous markers by RT-PCR, shown in Fig. 1. Overall, knockdown of COUP-TFII switches on the arterial EC markers, suggesting that COUP-TFII not only plays a role during development, but also is important for maintaining the venous identity in adult ECs.

Importantly, we found large number of chemokine/inflammatory genes are up-regulated as well as several genes involved in thrombosis ( supportingTable S1). To validate this finding, we performed RT-PCR analysis on several key genes known to be important for pro- or anti-atherogenic process. As shown in Fig. 1C, COUP-TFII knockdown lead to a decrease in TFPI2 (tissue factor pathway inhibitor 2), an anti-thrombotic gene, while increased the expression of VCAM-1 (vascular cell adhesion molecule), E-selectin, PAI-1 (plasminogen activator inhibitor 1), ECE (endothelin converting enzyme), ET-1 (endothelin 1), which are pro-atherogenic genes.

The overall pattern of pro-/anti-atherogenic gene expression suggests that the ECs with COUP-TFII knockdown switch to a more pro-atherogenic phenotype. To further validate this, we assessed inflammatory cytokine induced VCAM-1, the adhesion molecule involved in leukocyte-endothelial interaction in early atherosclerosis and is essential for disease progression^16^. As shown in Fig. 1D, COUP-TFII knockdown increased VCAM-1 expression at the basal level and also under low dose TNF-α stimulation. These data suggests that suppression of COUP-TFII in ECs makes them more sensitive to inflammatory stimuli. To further evaluate the functional significance of COUP-TFII knockdown in disease process, we performed leukocyte-endothelial adhesion assay. Shown in Fig. 1E, decreased COUP-TFII in ECs resulted in an increased THP-1 cell adhesion to EC surface under low dose of TNF-α (0.01 ng/ml) stimulation.

To further understand the functions of COUP-TFII in adult ECs, we made lentivirus over-expressing COUP-TFII and used it to generate confluent EC monolayer that stably expresses COUP-TFII. The transcriptional profiling analysis (supporting Table S3) revealed that COUP-TFII suppresses the known arterial marker expression, such as Ephrin-B2, Hes1/4. Multiple genes involved in angiogenesis/vasculogenesis are also suppressed, including Ephrin-A1, Ephrin-B3, HIF-2a, Sox4/17/18, Tbx1 and Vash1. Interestingly, many molecules involved in nerve development/axon guidance were also suppressed, such as Robo1, Netrin, Semaphorin and Plexin D1, which is consistent with the notion that nerve development guides the arterial differentiation^17^,^18^ whereas COUP-TFII suppresses these signals and leads to venous specification. Meanwhile, COUP-TFII up-regulated Lyve1 (lymphatic vessel endothelial hyaluronan receptor 1), a known marker for lymphatic ECs, consistent with the finding that lymphatic ECs are derived from venous ECs but not arterial ECs^19^,^20^.

**Figure 2:**
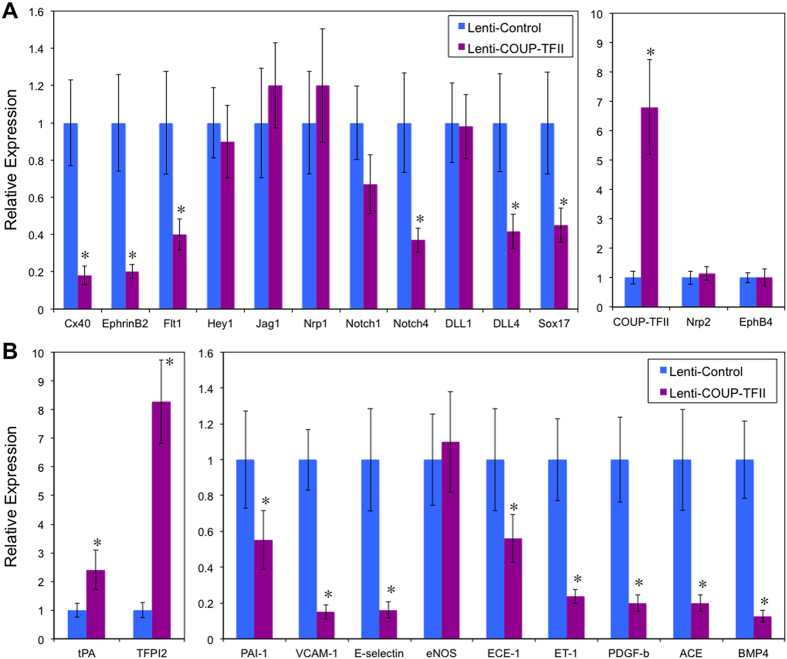
Over-expressing COUP-TFII suppresses arterial markers and promotes an athero-protective gene profile. (**A**,**B**) HSVECs were infected with lentivirus-COUP-TFII or control lentivirus (10 MOI), RNAs were isolated after confluent HSVEC monolayer is reached and all cells were permanently infected with lentivirus. Gene expressions were measured by Taqman-RT PCR and normalized to RNA 18S, n = 3, *p < 0.05.

COUP-TFII also suppresses or promotes distinct set of genes involved in the biosynthesis and remodeling of ECM. Besides various types of collagen, laminin, integrin and fibronectin, many ECM components and enzyme pathways are differentially regulated, including sulfatase, biglycan, versican, syndecan and matrix Gla protein (Supporting Table S3, S4). These data suggest that COUP-TFII may contribute to the distinct difference in the structural composition of arteries and veins. In agreement of the data that COUP-TFII knockdown increases inflammation and thrombosis, COUP-TFII over-expression suppressed inflammation and thrombotic gene profile (supporting Table S3) (*e.g*., VCAM-1, von Willebrand factor, thrombospondin, multimerin), and increased anti-thrombotic gene expression (supporting Table S4). We did additional validation using RT-PCR on selected genes. Shown in Fig. 2B, COUP-TFII increased tPA (tissue plasminogen activator) and TFPI2, both of which are anti-thrombotic, and suppressed the expression of PAI-1, VCAM-1, E-selectin, ET-1, ECE-1, PDGF-β (platelet derived growth factor beta), ACE (angiotensin converting enzyme), BMP4 (bone morphogenetic protein 4), all of which are pro-atherogenic genes.

**Figure 3:**
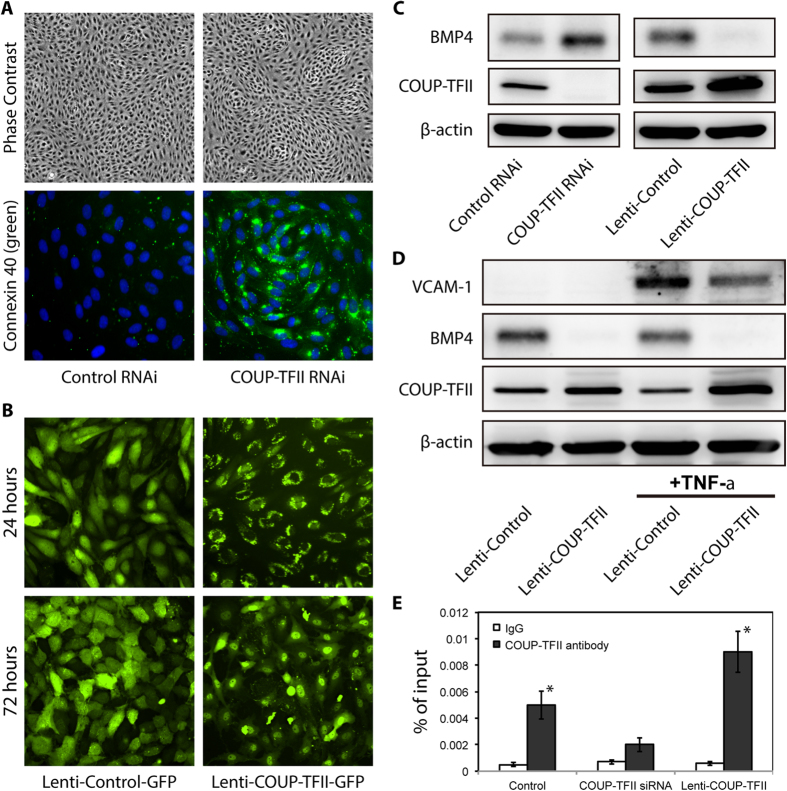
COUP-TFII regulates Cx40, BMP4 and VCAM-1 at protein level. (**A**) HSVECs were transfected with COUP-TFII or Control RNAi, and Cx40 was detected with immunofluorescent staining. (**B**) Fluorescent images of HSVECs transfected with lenti-Control-GFP or lenti-COUP-TFII-GFP. (**C**) Western blot analysis of BMP4 and COUP-TFII in HSVECs after COUP-TFII knockdown or over-expression. (**D**) HSVECs were treated with TNF-α (0.01 ng/ml) for 24 hours, western blot analysis of VCAM-1 expression was performed. (**E**) ChIP-PCR assays on cultured HSVECs using anti-COUP-TFII antibody (solid bar) or IgG (open bar). Bar graphs show enrichment of DNA fragments pulled down by COUP-TFII antibodies, *p < 0.01.

To further validate these findings, we examined the protein expression of COUP-TFII, BMP4 and VCAM-1. As shown in Fig. 3C, COUP-TFII RNAi completely knockdown the COUP-TFII at the protein level, whereas lentivirus over-expressing COUP-TFII increased the COUP-TFII protein, indicating the efficiency of the COUP-TFII knockdown and over-expression. Cytokine induced VCAM-1 expression demonstrates an opposite pattern of COUP-TFII expression (Fig. 3D): COUP-TFII suppressed TNF-α stimulated VCAM-1 protein, suggesting COUP-TFII has anti-inflammatory effect in ECs. Similarly, the BMP4 expression demonstrated opposite direction to COUP-TFII level (Fig. 3C): it increased in COUP-TFII knockdown and decreased in COUP-TFII over-expression, suggesting COUP-TFII is a negative regulator of BMP4. Because COUP-TFII demonstrates a strong regulation pattern on BMP4, we assessed whether COUP-TFII directly regulates BMP4 at the transcriptional level by ChIP (Chromatin Immunoprecipitation)-PCR. As shown in Fig. 3E, in both the control and COUP-TFII over-expression group, the immunoprecipitated chromatin using the COUP-TFII antibody demonstrates an enrichment of the BMP4 promoter compared to IgG control, suggesting that COUP-TFII may repress the BMP4 expression by binding directly to the BMP4 promoter region.

### Adult Arterial and Venous ECs Demonstrate Dramatic Differences in Gene Regulation Patterns and Response to Inflammatory Stimuli

**Figure 4:**
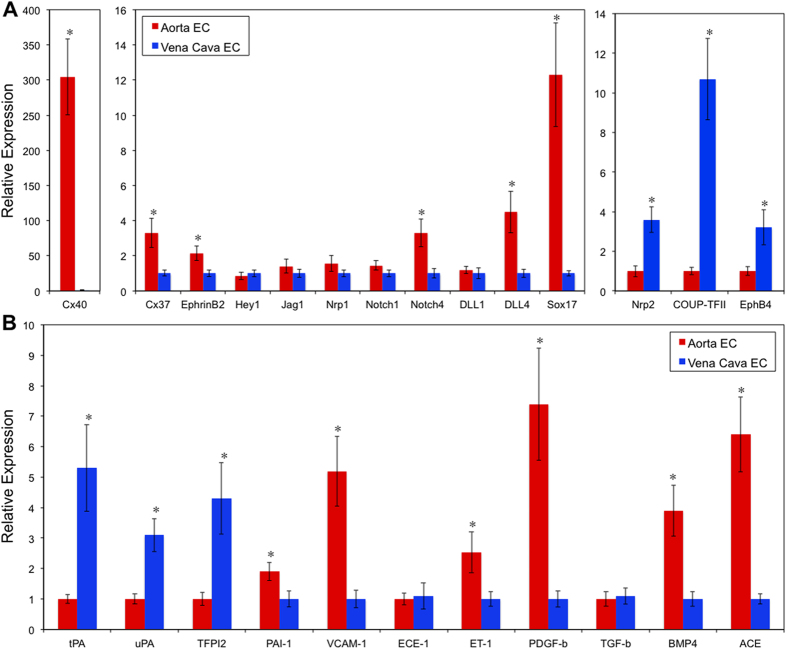
*In vivo* arterial and venous ECs demonstrate distinct profiles of certain molecular markers (A) and atherosclerosis related genes (B). RNA was isolated from the endothelium layer of mouse aorta and vena cava. Gene expression was analyzed by Taqman RT-PCR (n = 3), *p < 0.05.

To analyze the *in vivo* gene expression patterns, we developed the techniques to freshly isolate pure EC RNA (smooth muscle cell contamination <1%, supporting Figure S5) directly from intact mouse aorta and vena cava according to protocol approved by Albany Medical College’s Institutional Animal Care and Use Committee (IACUC). Analysis of transcriptional profiles showed that the fresh isolated EC RNA was able to re-confirm many of the known arterial venous marker expression patterns (Supporting Table S5, S6, Fig. 4A)^21^. We identified many new targets that are significantly expressed in arterial or venous ECs (Supporting Table S5, S6, Supporting Figure S1). The differences are dramatic; some of the genes are expressed more than 10–100 fold in arterial or venous ECs. Noticeable targets include Cx30/40, Adra2a (adrenergic receptor, alpha 2a), Abpd (androgen binding protein delta), Slit1, EphB6, Sox13/17, Fibulin 2, Ephrin-A5, Dlx5, which are highly expressed in artery, and Nppa/c (natriuretic peptide A & C), Tbx5, Claudin 11, Wnt2/9b, Apelin and COUP-TFII, which are highly expressed in vein. Importantly, we were able to confirm the pattern of COUP-TFII regulation related to thrombosis/atherogenesis: the expression of anti-thrombotic molecules tPA, TFPI2, uPA (urokinase-type plasminogen activator) is higher in vein, whereas pro-atherogenic molecules PAI-1, VCAM-1, ET-1, PDGF-α/β, BMP4 and ACE are higher in artery (Fig. 4B)

**Figure 5:**
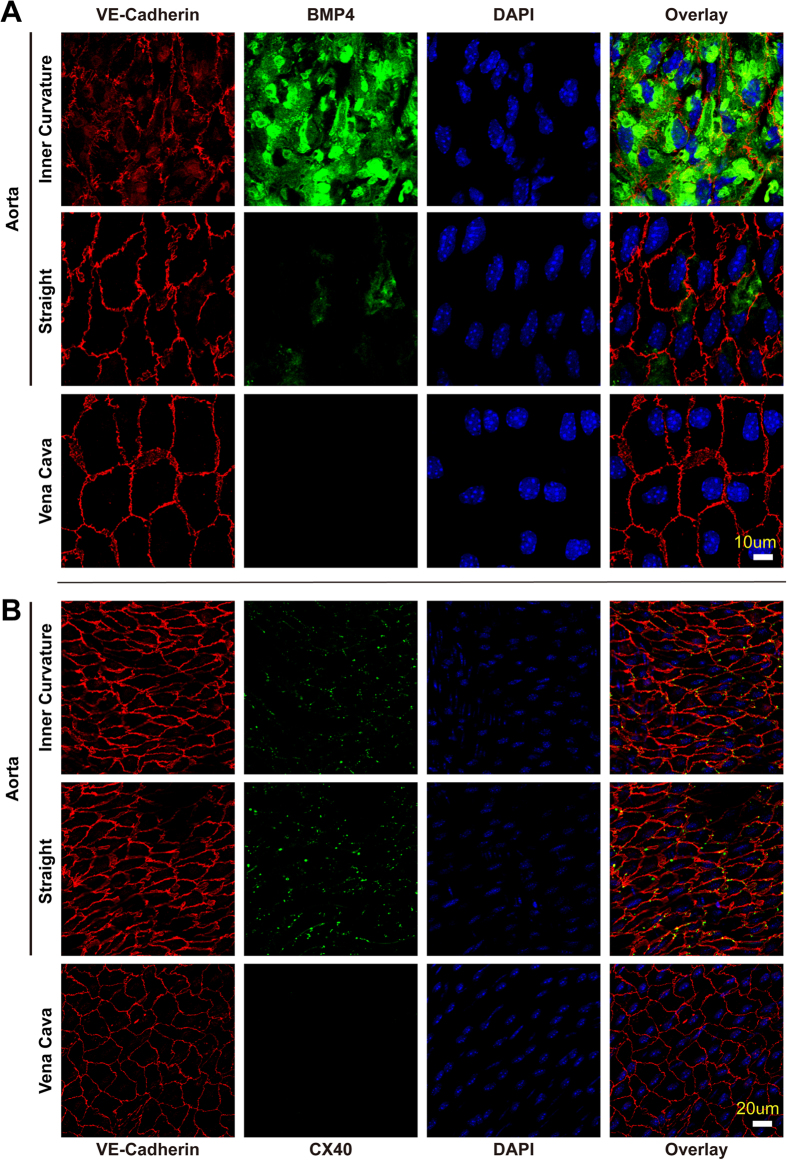
BMP4 and Cx40 protein is expressed in mouse arteries but not in veins. *en face* confocal immunofluorescent images of BMP4 (**A**) and Cx40 (**B**) expression in the endothelium layer of mouse aorta (straight portion and curvature) and vena cava.

One striking finding is gap junction protein Cx40, which has ~300 fold higher expression in arterial than venous ECs (Fig. 4A). The regulation by COUP-TFII is also consistent with this finding: COUP-TFII knockdown lead to a dramatic induction (>160 fold) of Cx40 (Fig. 1A), whereas it suppressed Cx40 when COUP-TFII is over-expressed (Fig. 2A). As shown in Fig. 3A, cultured HSVEC has very little Cx40 expression, which increased dramatically upon COUP-TFII knockdown. The *en face* immunofluorescent staining of mouse aorta and vena cava (Fig. 5B) further confirm this: Cx40 is completely absent in ECs of vena cava while it is abundantly located in the cell-cell junctions in both the straight and curvature portion of the aorta. Taken together, these data suggest that COUP-TFII is a strong regulator of gap junction protein Cx40 expression.

Using *en face* immunofluorescent staining technique, we also confirmed the BMP4 expression in artery and vein, and found BMP4 only appeared in the endothelial layer but not in the smooth muscle layer of the aorta (supporting Figure S2). As shown in Fig. 5A, BMP4 is highly expressed in the ECs of aortic curvature, and the expression is lower in the straight portion but clearly visible in some cells. In contrast, BMP4 is completely absent in the vena cava. This pattern of expression is consistent with the *in vivo* gene expression data and also the *in vitro* COUP-TFII regulation patterns, suggesting that COUP-TFII is a strong negative regulator of the BMP4 expression.

**Figure 6:**
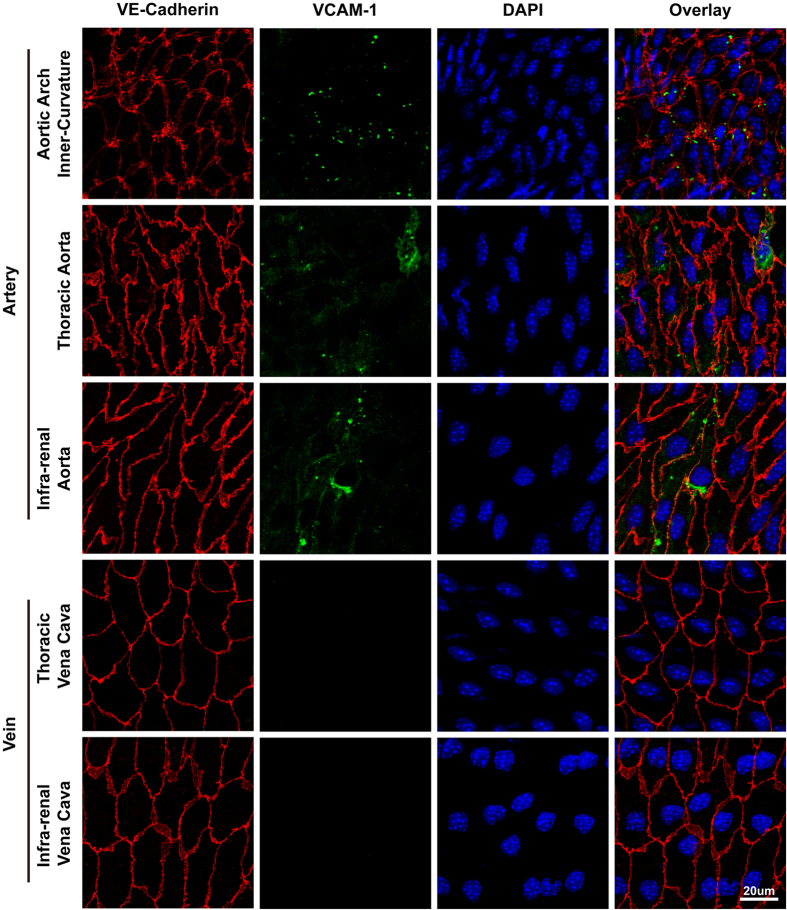
VCAM-1 is expressed in arteries but not veins in response to TNF-a stimulation. TNF-a was injected into mice for 16 hours. *en face* confocal immunofluorescent images of VCAM-1 expression in the endothelium layer of mouse arteries (inner-curvature of aortic arch, thoracic aorta, infra-renal aorta) and vena cava (thoracic vena cava and infra-renal vena cava).

To further assess the phenotypic differences of adult arterial and venous ECs, we examined the cytokine induced adhesion molecule VCAM-1 expression, which has been demonstrated to play an essential role in the initiation and progression of atherosclerosis^16^. TNF-a was injected into the mice as the inflammatory stimuli, and vascular expression of VCAM-1 was assessed via *en face* confocal immunofluorescent staining. As shown in Fig. 6, VCAM-1 staining is obvious across various parts of the arteries. In the inner curvature of the aortic arch where blood flow is disturbed, VCAM-1 appears as multiple punctuated staining throughout most regions, while the staining is more diffused and occurs in a few isolated cells in the thoracic aorta and infra-renal aorta. In contrast, VCAM-1 staining is completely absent in the thoracic vena cava and infra-renal vena cava, suggesting that adult artery and vein ECs have very different response to inflammatory stimuli.

**Figure 7:**
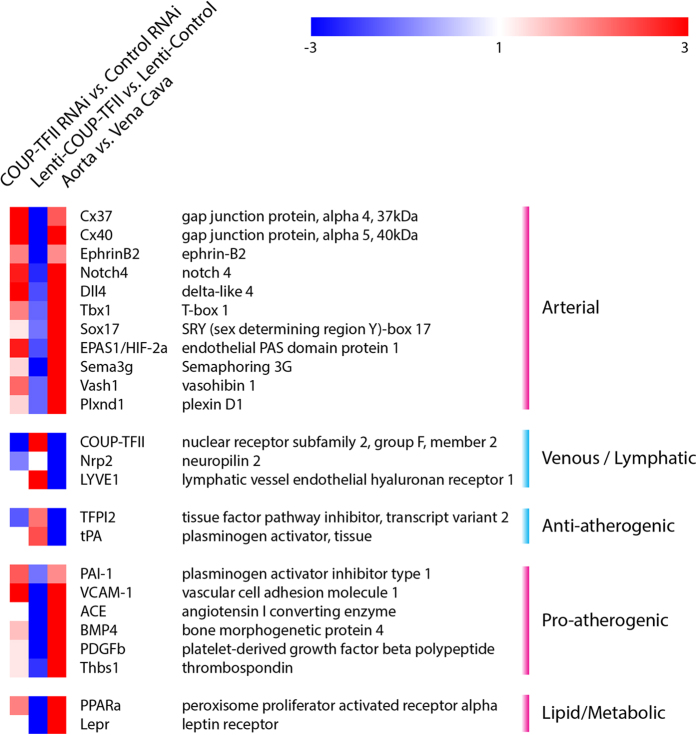
COUP-TFII controls the differential expression pattern of selected genes in artery *vs*. vein. Patterns of selected gene expression in COUP-TFII knockdown, COUP-TFII over-expression and *in vivo* arterial and venous ECs are color-coded. Genes that demonstrate consistent patterns of *in vitro* regulation (*either* knockdown *or* over-expression) with those *in vivo* expression patterns are selected.

To identify differentially expressed genes in arteries and veins that are also regulated by COUP-TFII, we compiled the list of genes that demonstrated consistency between *in vitro* COUP-TFII regulation and *in vivo* gene expression, and listed them in Fig. 7. Many arterial and venous genes are regulated by COUP-TFII, such as Cx37/40, Ephrin-B2, Notch4, Dll4, Tbx1, Sox17, HIF-2a, Sema3g, Vash1, Plxnd1, Nrp2 and Lyve1. Importantly, the selected anti- and pro-atherogenic genes are differentially expressed *in vivo* and they are also regulated by COUP-TFII: TFPI2 and tPA are higher in venous ECs while PAI-1, VCAM-1, ACE, BMP4, PDGFβ and Thrombospondin are higher in arterial ECs. Similar pattern of expression also includes genes in lipid/metabolic pathway such as PPAR-α and leptin receptor. Overall, arterial ECs demonstrated a more pro-atherogenic profile than venous ECs, and this difference is consistent with the *in vitro* COUP-TFII regulation data.

### COUP-TFII Regulates Endothelial-To-Mesenchymal Transition And Osteogenic Programs

**Figure 8:**
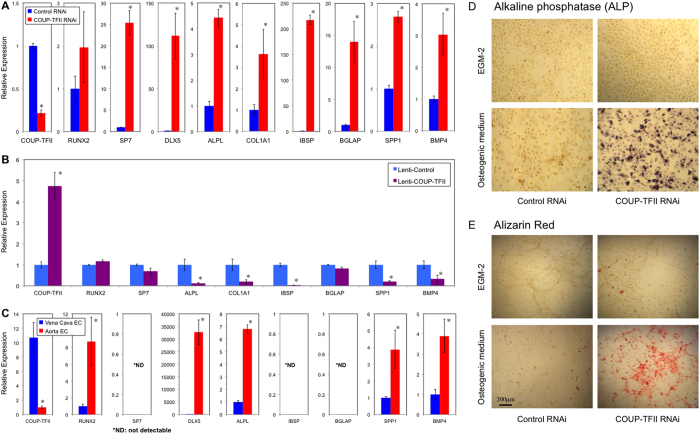
COUP-TFII negatively controls the osteogenic potential of ECs. (**A**) HSVECs transfected with Control or COUP-TFII RNAi were cultured under osteogenic differentiation medium for 7 days, and osteogenic related genes were assayed by Taqman RT-PCR (n = 3), *p < 0.05. (**B**) HSVECs transfected with lenti-Control or lenti-COUP-TFII were cultured under osteogenic differentiation medium for 7 days, and osteogenic related genes were assayed by Taqman RT-PCR (n = 3), *p < 0.05. (**C**) *In vivo* arterial and venous ECs demonstrate distinct osteogenic gene profile. RNA was isolated from the endothelium layer of mouse aorta and vena cava. Gene expression was analyzed by Taqman RT-PCR (n = 3), *p < 0.05, *ND: expression level is not detectable. (**D**,**E**) Alkaline Phosphatase and Alizarin Red staining of HSVECs after 7 days of culture under osteogenic differentiation medium.

**Figure 9:**
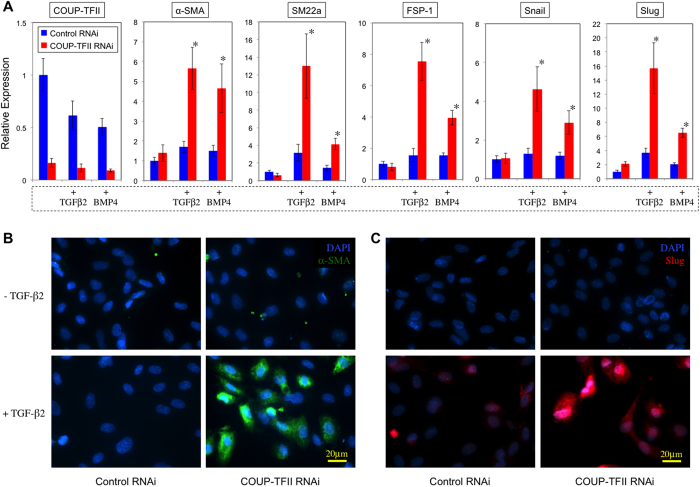
COUP-TFII negatively regulates EndMT. (**A**) HSVECs transfected with Control or COUP-TFII RNAi were cultured under EGM-2 (Control), or endothelial medium without VEGF and with TGFβ2 (10 ng/ml) or BMP4 (10 ng/ml) for 7 days, and gene expression was assayed by Taqman RT-PCR (n = 3), *p < 0.05. (**B**) Immunofluorescent staining of α-SMA. (**C**) Immunofluorescent staining of Slug.

Because several genes involved in bone homeostasis were regulated by COUP-TFII or showed differential expression in artery and vein (Supporting Table S3, S5), such as BMP4, MGP (matrix Gla protein) which is involved in calcium regulation and Dlx5 (distal-less homeobox 5) which acts as the early BMP-responsive transcriptional activator needed for osteoblast differentiation, we hypothesize that COUP-TFII regulates the osteogenic potential of ECs. To investigate the functional significance of COUP-TFII in this process, we treated the cells with BMP4 and switched the culture conditions to osteogenic medium, and assessed the osteogenic related programs in ECs with COUP-TFII knockdown and over-expression. Shown in Fig. 8A, in COUP-TFII knockdown condition, ECs readily undergo osteogenic differentiation with dramatically up-regulated genes involved in bone formation, such as Sp7 (osterix), Dlx5, ALPL (alkaline phosphatase), COL1A1 (collagen, type I, alpha 1), IBSP (integrin-binding sialoprotein), BGLAP (bone gamma-carboxyglutamate (gla) protein *or* osteocalcin), SPP1 (secreted phosphoprotein 1 *or* osteopontin) and BMP4. The effect is so significant that some of them have more than 10 or 100 fold of induction, suggesting that COUP-TFII in ECs acts as a strong repressor of its osteogenic potential in normal condition, and upon removal, osteogenic programs are greatly increased. Consistent with this, over-expression of COUP-TFII suppressed these genes: ALPL, COL1A1, IBSP, SPP1, BMP4 (Fig. 8B). Furthermore, biochemical assays demonstrated significant induction of alkaline phosphatase activity and calcium deposition in ECs cultured under osteogenic medium when COUP-TFII is knockdown (Fig. 8D,E). To further validate whether these osteogenic genes are also differentially expressed *in vivo*, we confirmed their expression pattern in ECs of mouse aorta and vena cava, and found that the expression of RUNX2, DLX5, ALPL, SPP1 and BMP4 are significantly higher in aortic ECs than vena cava ECs (Fig. 8C). Because upregulation of the osteogenic programs in ECs involves TGFβ/BMP signaling and the subsequent osteogenic differentiation, it is possible that TGFβ/BMP induced endothelial-to-mesenchymal transition (EndMT) is also affected. To test whether COUP-TFII regulates the EndMT, we cultured ECs under TGFβ2 treatment, which is a known inducer of EndMT. Transcriptional profile analysis revealed that several critical genes involved in the EndMT process are significantly induced when COUP-TFII is knockdown (Supporting Table S7). These changes collectively suggest an enhanced EndMT process^22^. Gene ontology enrichment analysis revealed that TGFβ pathway is particularly affected by COUP-TFII, including SMAD6, FST (follistatin), INHBA (inhibin, beta A, a subunit of activin and inhibin), TGFβ2 and TGFBI (transforming growth factor, beta-induced, 68 kDa) (Supporting Table S7). The most significantly up-regulated gene is TGFBI, which plays a role in cell-collagen interactions and is implicated in the endochondrial bone formation in cartilage. Shown in Fig. 9A, TGFβ2 or BMP4 treatment is a very weak inducer of EndMT in HSVECs. However, when COUP-TFII is knockdown by RNAi, the EndMT is significantly induced, demonstrated by increased expression of α-SMA, SM22α, FSP-1, Snail and Slug (Fig. 9A–C). Taken together, these data suggest that COUP-TFII is a strong regulator of the TGFβ pathway and TGFβ-induced EndMT in ECs.

### Hemodynamic Flow Is Not The Main Factor That Determines The Functional Difference Between Arterial And Venous ECs

Because hemodynamic flow in artery and vein may contribute to the differential gene expression patterns, we investigated whether arterial or venous flow regulates the selected arterial venous markers and atherogenesis related genes. Using a dynamic flow system, we re-created arterial and venous shear stress waveforms from prototypical flow patterns observed in human aorta and saphenous vein, and applied them to cultured HSVEC. Surprisingly, we found that there is only limited response to arterial flow in the selected arterial and venous markers (Supporting Figure S3). Among all of them, only Cx40 and Hey1 showed 4 ~ 5 folds of induction by arterial flow, while venous flow is very similar to static culture. This is in sharp contrast to the hundreds of folds regulation of Cx40 *in vivo* and COUP-TFII knockdown data, suggesting flow is not the dominant factor that controls these markers.

Arterial flow up-regulated anti-thrombotic genes tPA and TFPI2 while down-regulated pro-atherogenic genes PAI-1, VCAM-1, E-selectin, ET-1, ACE, BMP4 and PDGF-β, which is consistent with previous knowledge that pulsatile flow in artery leads to a more anti-atherogenic phenotype^23^. This result is exactly the opposite of the gene expression pattern observed in arteries and veins *in vivo*: arterial ECs display a more pro-atherogenic phenotype with lower expression of tPA, TFPI2 and higher expression of PAI-1, VCAM-1, E-selectin, ET-1, ACE, BMP4 and PDGF-β. Taken together, these data suggest that hemodynamic flow is not the primary factor that is responsible for the observed gene expression patterns, but the endogenous genetic/epigenetic programs of arteries and veins are more likely the driving force for these differences.

## Discussion

In this study we investigated the role of COUP-TFII in regulating adult EC functions, focusing on their significance in vascular diseases. We aim to examine the multifaceted roles of COUP-TFII in vascular disease by comprehensive analysis of EC gene expression and phenotypes using a model of COUP-TFII knockdown and over-expression in adult human ECs, and subsequent validation in adult blood vessels in animal models. We have identified differential patterns of gene expression in adult arterial and venous ECs, and confirmed that some of those genes are under the control of COUP-TFII. Many of the differentially regulated genes have pathophysiological functions in atherosclerosis initiation and/or progression, thus demonstrating the importance of COUP-TFII in regulating adult EC functions. In particular, venous ECs with COUP-TFII knockdown acquire some of the arterial gene expression patterns and a pro-inflammatory phenotype, expressing several important molecules in atherogenesis and lowering several anti-thrombotic genes. It also elicited an enhanced cytokine-inducible cell surface expression of the atherosclerosis-associated adhesion molecule VCAM-1 and leukocyte adhesion to EC surface. Importantly, we also discovered that COUP-TFII not only regulates the expression of BMP4 and TGFβ2, but also affects multiple signaling molecules in the BMP4/TGFβ pathway. BMP signaling is known to promote vascular calcification, and others have reported that limiting vascular BMP signaling decreases both atherosclerotic lesion and diabetic medial calcification^24^. In addition, we have observed an enhanced EndMT and strikingly increased osteogenic potential of ECs when COUP-TFII is knockdown. Consistently, over-expression of COUP-TFII resulted in the totally opposite effects. Finally, we validated these findings in ECs of mouse aorta and vena cava *in vivo*. Taken together, these data strongly implicate the modulation of endothelial phenotype by venous marker COUP-TFII in the vascular system.

Specification of vascular progenitors into arterial and venous ECs is an essential process of vascular development^9^,^25^,^26^,^27^,^28^,^29^,^30^,^31^,^32^. Several signaling pathways are involved in the arterial-venous differentiation, among which the Notch signaling plays a central role^29^,^30^,^33^,^34^. Blood flow is another critical factor in determining arterial differentiation^35^. Lack of Notch activation or blood flow was thought to be the reason for venous differentiation. However, the discovery of COUP-TFII demonstrated that COUP-TFII functions as a key player that confers vein identity by repressing Notch signaling^9^. Recent study also confirmed that over-expression of COUP-TFII suppresses Notch pathway in cultured human umbilical artery endothelial cells^15^. Our data demonstrated that COUP-TFII not only plays a role in regulating arterial venous markers, but also control important functions in adult ECs, suggesting that these developmental programs also contribute toward the adult blood vessel functions in the pathogenesis of vascular diseases.

Although blood flow plays an important role in arterial EC specification during early vascular development, arterial markers in adult blood vessel are less responsive to hemodynamic flow after the vasculature is fully developed. When placed in arterial circulation, vein graft undergoes “arterialization”^36^,^37^ in which smooth muscle cells adapted to higher pressure and cyclic strain in responses to the new milieu. In ECs, venous identity (EphB4) is lost during this process, but arterial identities (Ephrin-B2, DLL4, Notch4) are not gained, suggesting an incomplete adaptation of vein graft in adult patients^38^,^39^. Our data also confirmed that the arterial-venous markers in adult ECs are less responsive to flow condition. We found that adult EC has very limited response to flow, and most arterial and venous markers do not change except Cx40 and Hey1. The relatively small change of Cx40 is also far from the hundreds fold differences observed in artery *vs*. vein *in vivo*. This result demonstrates that adult ECs lose some of their plasticity and are not able to completely adapt to arterial circulatory conditions.

We have also identified many new molecular markers that are highly expressed in adult artery or vein. Some of these markers show more than 10–100 fold of differential expression, and therefore, can be used as a more robust marker to identify artery *vs*. vein than those with only 2 ~ 3 folds of difference. Surprisingly, some of the well-established arterial markers (Nrp1, Hey1, Jag1, Dll1) are not differentially expressed in adult arteries *vs*. veins, although they have been used extensively in the past as the proof of arterial differentiation in many developmental biology studies. It is likely that they appear as early arterial marker during vascular development, and then the difference disappears after maturation into adult blood vessel. Interestingly, although COUP-TFII controls many arterial venous markers, EphB4 seems not influenced by COUP-TFII at all. This suggests that other additional unknown endogenous programs are involved in venous specification besides COUP-TFII.

In arteries, atherosclerosis often develops in regions of disturbed flow, which promotes a pro-inflammatory phenotype of ECs^23^,^40^,^41^. In contrast, laminar pulsatile flow in straight portion of arteries leads to an anti-inflammatory and anti-thrombotic phenotype, therefore, is considered athero-protective^42^,^43^,^44^. Unlike in arteries, blood flow in veins is not pulsatile but phasic with low average wall shear stress (<1 dyn/cm^2^). Based on our previous extensive knowledge of flow-regulated EC functions, this type of flow is not considered “athero-protective” since it has very small influence on EC gene expression (compared to static control) whereas the influence of laminar pulsatile arterial shear is much larger. Despite this unfavorable hemodynamic condition, veins rarely develop certain types of vascular diseases such as atherosclerosis and vascular calcification, suggesting that endogenous genetic/epigenetic programs in veins protect them from these vascular diseases. Our data indicate arterial flow promotes an athero-protective phenotype while the ECs cultured under venous flow conditions exhibit a more athero-prone phenotype in comparison to arterial flow. However, the transcriptional profiling analysis in actual arteries and veins reveals a profile that is completely the opposite: arterial ECs demonstrate a more athero-prone profile and osteogenic program than venous ECs, which is more consistent with the pattern of COUP-TFII regulation. The *en face* VCAM-1 staining in blood vessels also confirmed that arterial ECs have a much stronger response to inflammatory stimuli by turn on VCAM-1 expression whereas venous ECs do not express VCAM-1 at all. VCAM-1 was reported to express at the arteries and post-capillary venules after cytokine stimulation^14^, but its expression in the major adult veins has not been examined in the past. Our finding that VCAM-1 is absent in large veins is very exciting. Because VCAM-1 is an essential adhesion molecule involved in atherosclerosis initiation and progress, the drastic difference in its expression in adult arteries and veins suggest that it may correlate with the low susceptibility of atherosclerosis in veins. Our data again confirmed that arterial ECs have a more athero-prone profile than venous ECs in response to inflammatory stimuli, which is consistent with the COUP-TFII regulated gene patterns but is totally the opposite to those predicted by hemodynamic flows. Taken together, these data suggest that hemodynamic flow is not the primary factor that determines the molecular divergences in artery and vein. Instead, the endogenous genetic/epigenetic programs (such as COUP-TFII) are stronger regulators that control these molecular and functional differences.

Vascular calcification is a frequent complication of vascular disease, such as diabetes mellitus, renal disease, and atherosclerosis. It is a regulated process with strong similarities to bone formation driven by osteo-progenitor cells in the media layer of blood vessel. Vascular EC represents a distinct cell lineage from osteo-progenitor cells. In healthy blood vessels and also in normal EC culture, the osteogenic program is usually absent and thus ECs do not contribute to the calcium deposition in healthy blood vessels. However, recent studies have demonstrated a role for the endothelium in vascular calcification^45^ in disease condition with high BMP activity. Under the stimulation of BMP4 (or TGFβ2), ECs can be converted into mesenchymal stem cell (MSC)-like cells through EndMT process and undergo osteogenic differentiation under osteogenic condition^46^. This leads to the up-regulation of series gene pathways similar to osteogenic differentiation of MSCs. EndMT has also been reported in ECs derived from the mitral valve leaflets^47^ and in HAECs *in vitro*^48^, which support an important role for the endothelium in the development of cardiovascular calcification. Interestingly, in animal models, the EndMT and multipotent cells were found to be restricted to the artery wall but not in the veins in these mice^45^. Our data reveal mechanistic insight into this difference that COUP-TFII negatively regulates the EndMT process. It also plays a strong role in suppressing the osteogenic potential and subsequent calcium deposition of adult ECs, suggesting that COUP-TFII may contribute to the different susceptibility of arteries and veins to calcification-related vascular diseases. The higher expression of RUNX2, ALPL, SPP1 and DLX5 in arterial ECs *in vivo* is also striking, indicating a stronger osteogenic potential of arteries. EndMT has been described in different pathologies, and is implicated in many disease situations such as cancer progression^49^, cardiac fibrosis^50^, vascular calcification^45^, and intimal hyperplasia during vein graft remodeling^51^. Our findings of COUP-TFII as the suppressor of EndMT process suggest that it can be used as a broad therapeutic target for a variety of EndMT-related disease conditions.

In summary, COUP-TFII, a transcription factor expressed higher in venous EC, plays multifaceted roles in determining the functional difference between arteries and veins. Multiple important gene pathways involved in vascular diseases are regulated by COUP-TFII. ECs with higher COUP-TFII expression confer a more anti-atherogenic phenotype and lower osteogenic potential, while lower COUP-TFII expression elicits a more pro-atherogenic phenotype and greater sensitivity to EndMT and osteogenic stimulation. Our findings provide new insights into the mechanism of arterial diseases and suggest potential roles of COUP-TFII in determining the distinctive susceptibilities of arteries and veins to vascular diseases.

## Additional Information

**How to cite this article**: Cui, X. *et al.* Venous Endothelial Marker COUP-TFII Regulates the Distinct Pathologic Potentials of Adult Arteries and Veins. *Sci. Rep.* **5**, 16193; doi: 10.1038/srep16193 (2015).

## Supplementary Material

Supplementary Information
