# Supplementary material for: Venous Endothelial Marker COUP-TFII Regulates the Distinct Pathologic Potentials of Adult Arteries and Veins
Source: Sci Rep. 2015 Nov 5;5:16193. doi: 10.1038/srep16193 (PMC4633649; doi:10.1038/srep16193)
Supplement: Supplementary Information [file srep16193-s1.doc]

# Supporting Information

# Venous Endothelial Marker COUP-TFII Regulates the Distinct Pathologic Potentials of Adult Arteries and Veins

Xiaofeng Cui^1,3*^, Yao Wei Lu^4*^, Vivian Lee^2,3^, Diana Kim^2,3^, Taylor Dorsey^2,3^, Qingjie Wang^5^, Young Lee^3^, Peter Vincent^4^, John Schwarz^4^, Guohao Dai^2,3**^

1. School of Chemistry, Chemical Engineering and Life Sciences, Wuhan University of Technology, Wuhan, China 430070.

2. Department of Biomedical Engineering, Rensselaer Polytechnic Institute, Troy, NY 12180, USA.

3. Center for Biotechnology and Interdisciplinary Studies, Rensselaer Polytechnic Institute, Troy, NY 12180, USA

4. The Center for Cardiovascular Sciences, Albany Medical College, Albany, NY 12208, USA.

5. Neural Stem Cell Institute, Rensselaer, NY 12144, USA.

*These authors contribute equally to the work.

**Corresponding author:

Guohao Dai, Ph.D.

Associate Professor

Department of Biomedical Engineering

Center for Biotechnology and Interdisciplinary Studies, Room 3123

Rensselaer Polytechnic Institute

110 8th Street

Troy, NY 12180

Phone: 518-276-4476

Fax: 518-276-3035

Email: daig@rpi.edu

# Supporting Information

### SI Materials and Methods

Apply Arterial And Venous Flow To Cultured ECs

To apply arterial and venous flow to cultured ECs, we used the dynamic flow system previously developed in the lab ^1^ to re-create the pulsatile shear stress waveform derived from typical human abdominal aorta and saphenous vein blood flow patterns. The dynamic flow system is under the computer control to enable accurate program of any shear stress waveform and applied it to cultured ECs. HSVECs were plated at an initial density of 50,000 cells/cm^2^ on 0.1% gelatin (Sigma) coated tissue culture plate and the cell culture plate was assembled in the dynamic flow system. Fully confluent EC monolayer was then exposed to the arterial or venous shear stress waveforms for 72 hours. Culture medium in the shear apparatus was replenished during the experiment at an exchange rate of 0.1 ml/min, and the enclosed environment was maintained at 37°C in a humidified 5% CO_2_/95% air atmosphere. For comparison, EC from the same passage were plated on an identical plastic plate assembly and incubated at 37°C in a 5% CO_2_/95% air atmosphere under static (no flow) conditions.

RNA Isolation and Real-Time PCR

RNA was isolated using RNeasy Plus Mini Kit (Qiagen) according to the manufacturer’s protocol. The concentration and quality of RNA was determined by NanoDrop (Thermo Scientific). Gene expression was measured by Quantitative Real-time PCR reactions using Taqman RT-PCR assays (Life Technologies) on StepOne Plus RT-PCR system (Life Technologies). The relative gene expression was normalized to 18S ribosomal RNA.

Microarray Hybridization and Statistical Methods for Microarray Data Analysis

For microarray analysis, three independent sets of experiments were performed. Microarray analysis was performed using the Human Whole Genome OneArray® v5 (Phalanx Biotech), which contains 30,275 oligonucleotides representing 29,187 human genome probes and 1,088 experimental control probes. RNA quality and integrity were determined utilizing an Agilent 2100 Bioanalyzer (Agilent Technologies) and a NanoDrop spectrophotometer (Thermo Scientific). Only high quality RNA, having a RIN of >6.0, and absorbance ratios A260/A280 >1.8 and A260/A230 >1.5, was utilized for further experimentation. RNA was converted to double-stranded cDNA and amplified using in vitro transcription that included amino-allyl UTP, and the cDNA product was subsequently conjugated with Cy5™ NHS ester (GEH Lifesciences). Fragmented cDNA was hybridized at 42 ⁰C overnight using the HybBag mixing system with 1X OneArray Hybridization Buffer (Phalanx Biotech), 0.01 mg/ml sheared salmon sperm DNA (Promega), at a concentration of 0.025 mg/ml labeled target. After hybridization, the arrays were washed according to the OneArray protocol.

Raw intensity signals for each microarray were captured using a Molecular Dynamics™ Axon 4100A scanner, measured using GenePixPro™ Software, and stored in GPR format. The data from all microarrays in each experimental set was then passed to Rosetta Resolver (Rosetta Biosoftware) for analysis. Testing was performed by combining replicates and performing statistical analyses using Rosetta Resolver. The Rosetta error model captures the variance-intensity relationship for various types of microarray technologies. This error model conservatively estimates intensity error and uses this value to stabilize the variance estimation. These technology-specific error models are designed and optimized for different microarray technologies, such as Affymetrix® and Agilent Technologies. Data from three independent experiments were analyzed, and the averaged ratio and *p*-value were obtained for each paired comparison. To decrease the false-positive rate associated with the multiple comparisons, the Bonferroni correction was applied to the analysis, gene regulations were considered statistically significant when *p* < 0.05/(total number of genes tested)=0.05/29,187.

Chromatin Immunoprecipitation (ChIP)－PCR

Confluent ECs under various experimental conditions were cross-linked by addition of 1% formaldehyde and subsequently harvested. Chromatin immunoprecipitation on the samples was performed as previously described ^2^, using mouse monoclonal anti-human COUP-TFII antibody (R&D Systems). Mouse IgG (Life Technologies) was used as negative control. DNA released from the precipitation was subjected to qPCR analysis for quantification of the presence of specific loci. The promoter region of human BMP-4 was analyzed by qPCR with the primers 5’- ACAGCTCCATCAGAGGCAGT -3’ and 5’- GAAACAGGCTGTGTGCAGAA -3’, which spans the 1.5kb upstream of the transcription start site of BMP4 gene. The relative expression was compared with the amplification of input chromatin not subjected to immunoprecipitation.

Transfection of siRNA Duplexes into HSVEC

siRNA to knockdown COUP-TFII and control siRNA were synthesized by Thermo Scientitic Bio. siRNA was transfected into HSVEC with Lipofectamine RNAiMAX (Life Technologies) and Opti-MEM (Life Technologies) at a final concentration of 10nM according to manufacturer’s instruction. RNA was isolated 72 hours after the siRNA transfection. The efficiency of knockdown was greater than 80% as assessed by both western blot and RT-PCR. To confirm the specificity of the siRNA knockdown, selected experiments were also performed using a different siRNA construct (Life Technologies) and an shRNA construct (Sigma Mission Lentivirus shRNA, Sigma).

Immunofluorescence Microscopy of Cellular Proteins

EC were rinsed in cold PBS and fixed in 4% paraformaldehyde for 10 minutes and then permeabilized in 0.1% Triton X-100 for 1 minutes at room temperature. To reduce non-specific immunoglobulin binding, cells were pre-incubated in 1% BSA for 1 hour. For Cx40 staining, cells were incubated with anti-Cx40 antibody (1:200, rabbit polyclonal IgG, Alpha Diagnostic International) for 1 hour at room temperature, rinsed with 0.1% BSA in PBS, and incubated for 30 minutes with 2% goat serum in PBS. Cells were incubated with Alexa Fluor 488 goat anti-rabbit (1:500, Life Technologies) for 1 hour at room temperature, rinsed in PBS, and mounted using Gel-Mount (Biomeda). Immunostaining of Slug and α-SMA was done using the same method with anti-Slug antibody (1:200, rabbit polyclonal IgG, Cell Signaling) and anti-α-SMA antibody (1:500, mouse monoclonal IgG, Sigma).

Western Blot Analysis of Protein Expression

Cells growing on 6-well plates were rinsed with ice-cold PBS and scraped after lysis with 300ul Laemmli buffer containing complete protease inhibitor mixture (Roche Applied Science), PhosSTOP phosphatase inhibitor mixture (Roche Applied Science), and subsequently boil for 5 minutes. 30ul of the cell lysate from each condition were then loaded on standard SDS-PAGE gels and transferred to nitrocellulose membranes. Immunoblots were performed by blocking the membranes with 3% non-fat milk in TBS and incubate at 4°C overnight with any of the following antibodies: anti-BMP4 (1:500, mouse monoclonal IgG, Santa Cruz Biotechnology), anti-COUP-TFII (1:250, mouse monoclonal IgG, R&D Systems), anti-VCAM-1 (1:250, mouse monoclonal IgG, BD Biosciences) and anti-β-actin antibody (1:5000, mouse monoclonal IgG, Sigma-Aldrich). Secondary anti-mouse antibody conjugated with HRP (Jackson ImmunoResearch Laboratories) were incubated for 2 hour at room temperature. Membrane were developed using SuperSignal West Pico or Femto chemiluminescent substrate (Pierce) and Fujifilm LAS-3000 imaging system.

Leukocyte Adhesion Assays

THP-1 cells (ATCC) were cultured in suspension in tissue culture flask with RPMI-1640 Medium (ATCC) supplemented with 50uM β-mercaptoethanol and 10% fetal bovine serum, and passaged according to manufacturer’s protocol. One million THP-1 cells were labeled with Cell Tracker Green (Life Technologies) as instructed by the manufacturer. To evaluate the leukocyte-endothelial adhesion, confluent HSVEC monolayer was treated with TNF-α (0.01ng/ml) for 24 hours. HSVECs were washed twice with PBS to remove cell debris, and the 5 ml of THP-1 suspension (1 million cells total) was added to the cells. The dish was place on a horizontal rotator at approximately 60 rpm for 10 minutes at room temperature to allow binding. Monolayers were then washed gently with PBS 3 times, and then visualized under a phase-contrast microscope with a 488 filter (to visualize labeled THP-1 cells). For quantification of bound THP-1 cells, the number of cells in each field is counted and averaged from 10 randomly chosen field.

### Supporting Tables

* Data from three independent experiments were analyzed by statistical methods (see *Supporting* Materials and Methods). To decrease the false-positive rate associated with the multiple comparisons, the Bonferroni correction of *p*-value was applied. The listed genes have at least two folds changes, and have a *p*< 0.05/(total number of genes tested)=0.05/29,187.

### Table S1. Selected Genes That Are Up-Regulated After COUP-TFII Knockdown

| **Category** | | **Gene Name** | **Gene Symbol** | **Fold Change** | **Accession #** |
| --- | --- | --- | --- | --- | --- |
| *Vascular Development / Arterial-Venous Differentiation* | | | |  |  |
|  | hairy/enhancer-of-split related with YRPW motif 2 | | HEY2 | 6.7 | NM_012259.2 |
|  | semaphorin 7A, GPI membrane anchor | | SEMA7a | 6.0 | NM_003612.3 |
|  | delta-like 4 | | DLL4 | 4.1 | NM_019074.3 |
|  | hairy and enhancer of split 4 | | HES4 | 3.4 | NM_021170.3 |
|  | hairy/enhancer-of-split related with YRPW motif 1 | | HEY1 | 3.1 | NM_012258.3 |
|  | fms-related tyrosine kinase 1 | | FLT1/VEGFR-1 | 2.9 | NM_002019.4 |
|  | endothelial PAS domain protein 1 | | EPAS1/HIF-2a | 2.8 | NM_001430.4 |
|  | notch 4 | | Notch4 | 2.8 | NM_004557.3 |
|  | forkhead box C2 | | FOXC2 | 2.3 | NM_005251.2 |
|  | delta-like 1 | | DLL1 | 2.2 | NM_005618.3 |
|  | vasohibin 1 | | VASH1 | 2.2 | NM_014909.4 |
|  | ephrin-A1 | | Ephrin-A1 | 2.1 | NM_004428.2 |
|  | TEK tyrosine kinase, endothelial | | TIE-2 | 2.1 | NM_000459.3 |
|  | EPH receptor A4 | | EphA4 | 2.0 | NM_004438.3 |
|  | ephrin-B2 | | Ephrin-B2 | 2.0 | NM_004093.3 |
|  | jagged 1 | | JAG1 | 2.0 | NM_000214.2 |
| *Inflammation* | | | |  |  |
|  | chemokine (C-X-C motif) ligand 10 | | CXCL10 | 36 | NM_001565.2 |
|  | chemokine (C-X-C motif) ligand 11 | | CXCL11 | 10 | NM_005409.4 |
|  | tumor necrosis factor (ligand) superfamily, member 10 | | TNFSF10 | 3.9 | NR_033994.1 |
|  | chemokine (C-C motif) ligand 5 | | CCL5 | 3.8 | NM_002985.2 |
|  | interleukin 15 receptor, alpha | | IL15RA | 3.4 | NM_002189.3 |
|  | chemokine (C-C motif) ligand 5 | | CCL5 | 3.3 | NM_002985.2 |
|  | chemokine (C-X3-C motif) ligand 1 | | CX3CL1 | 3.2 | NM_002996.3 |
|  | interleukin 1, alpha | | IL1A | 2.3 | NM_000575.3 |
|  | chemokine (C-X-C motif) ligand 12 | | CXCL12 | 2.2 | NM_000609.5 |
|  | interleukin 3 receptor, alpha (low affinity) | | IL3RA | 2.0 | NM_002183.2 |
|  | tumor necrosis factor receptor superfamily, member 4 | | TNFRSF4 | 2.0 | NM_003327.3 |
|  | chemokine (C-X-C motif) ligand 2 | | CXCL2 | 2.0 | NM_002089.3 |
| *Extracellular Matrix Biosynthesis* | | | |  |  |
|  | collagen, type IV, alpha 2 | | [COL4A2](http://www.ncbi.nlm.nih.gov/sites/entrez?db=gene&cmd=search&term=COL4A2) | 2.3 | [NM_001846.2](http://www.ncbi.nlm.nih.gov/entrez/viewer.cgi?val=NM_001846.2) |
|  | integrin, alpha 3 | | [ITGA3](http://www.ncbi.nlm.nih.gov/sites/entrez?db=gene&cmd=search&term=ITGA3) | 2.3 | NM_005501.2 |
|  | laminin, alpha 5 | | [LAMA5](http://www.ncbi.nlm.nih.gov/sites/entrez?db=gene&cmd=search&term=LAMA5) | 2.2 | [NM_005560.3](http://www.ncbi.nlm.nih.gov/entrez/viewer.cgi?val=NM_005560.3) |
| *Thrombosis / Vasomotor Function* | | | |  |  |
|  | serpin peptidase inhibitor, clade B, member 1 | | SERPINB1 | 3.7 | [NM_030666.2](http://www.ncbi.nlm.nih.gov/entrez/viewer.cgi?val=NM_030666.2) |
|  | serpin peptidase inhibitor, clade E (nexin, plasminogen activator inhibitor type 1), member 2 | | SERPINE2/PAI-1 | 2.4 | NM_006216.3 |
|  | endothelin converting enzyme 1 | | [ECE-1](http://www.ncbi.nlm.nih.gov/sites/entrez?db=gene&cmd=search&term=ECE1) | 2.0 | NM_001397.2 |
| *Lipid / Metabolic Pathways* | | | |  |  |
|  | apolipoprotein L, 3 | | ApoL3 | 2.7 | NR_027835.1 |
|  | ATP-binding cassette, sub-family G, member 1 | | ABCG1 | 2.1 | NM_207629.1 |
| *Junction Proteins* | | | |  |  |
|  | gap junction protein, alpha 5, 40kDa | | Cx40 | 6.2 | NM_181703.2 |
|  | gap junction protein, alpha 4, 37kDa | | Cx37 | 3.4 | [NM_002060.2](http://www.ncbi.nlm.nih.gov/entrez/viewer.cgi?val=NM_002060.2) |

### Table S2. Selected Genes That Are Down-Regulated After COUP-TFII Knockdown

| **Category** | **Gene Name** | **Gene Symbol** | **Fold Change** | **Accession #** |
| --- | --- | --- | --- | --- |
| *Vascular Development / Arterial-Venous Differentiation* | | |  |  |
|  | nuclear receptor subfamily 2, group F, member 2 | COUP-TFII | -5.3 | NM_021005.3 |
|  | neuropilin 2 | Nrp2 | -2.0 | NM_201266.1 |
| *Thrombosis / Vasomotor Function* | | |  |  |
|  | tissue factor pathway inhibitor 2 | TFPI2 | -2.3 | NM_006528.2 |
|  | prostaglandin reductase 1 | PTGR1 | -2.0 | NM_012212.3 |
| *Extracellular Matrix* | | |  |  |
|  | syndecan 1 | SDC1 | -2.2 | NM_002997.4 |
| *Lipid / Metabolic Pathways* | | |  |  |
|  | low density lipoprotein receptor | LDLR | -4.3 | NM_000527.4 |

### Table S3. Selected Genes That Are Down-Regulated After Over Expressing COUP-TFII

| **Category** | **Gene Name** | **Gene Symbol** | **Fold Change** | **Accession #** |
| --- | --- | --- | --- | --- |
| *Vascular Development / Arterial-Venous Differentiation* | | |  |  |
|  | roundabout, axon guidance receptor, homolog 1 | ROBO1 | -102 | NM_133631.3 |
|  | semaphorin 3G | SEMA3G | -10.8 | NM_020163.1 |
|  | netrin 4 | NTN4 | -6.3 | NM_021229.3 |
|  | ephrin-A1 | ephrin-A1 | -5.1 | NM_004428.2 |
|  | ephrin-B2 | ephrin-B2 | -3.9 | NM_004093.3 |
|  | hairy and enhancer of split 4 | HES4 | -3.6 | NM_021170.3 |
|  | jagged 2 | JAG2 | -3.3 | NM_145159.1 |
|  | ephrin-B3 | ephrin-B3 | -3.2 | NM_001406.3 |
|  | semaphorin 6C | SEMA6C | -3.0 | NM_030913 |
|  | hairy and enhancer of split 1 | HES1 | -2.6 | NM_005524.3 |
|  | endothelial PAS domain protein 1 | EPAS1/HIF-2a | -2.4 | NM_001430.4 |
|  | SRY (sex determining region Y)-box 4 | SOX4 | -2.3 | NM_003107.2 |
|  | plexin D1 | PLXND1 | -2.2 | NM_015103.2 |
|  | SRY (sex determining region Y)-box 18 | SOX18 | -2.2 | NM_018419.2 |
|  | T-box 1 | TBX1 | -2.2 | NM_080647.1 |
|  | vasohibin 1 | VASH1 | -2.2 | NM_014909.4 |
|  | SRY (sex determining region Y)-box 17 | SOX17 | -2.1 | NM_022454.3 |
| *Inflammation* | | |  |  |
|  | vascular cell adhesion molecule 1 | VCAM-1 | -9.2 | NM_080682.2 |
|  | chemokine (C-C motif) ligand 2 | CCL2 | -5.9 | NM_002982.3 |
|  | chemokine (C-C motif) ligand 23 | CCL23 | -4.4 | NM_145898.1 |
|  | chemokine (C-C motif) ligand 14 | CCL14 | -3.6 | NM_032962.4 |
|  | tumor necrosis factor (ligand) superfamily, member 10 | TNFSF10 | -3.6 | NR_033994.1 |
|  | chemokine (C-C motif) ligand 11 | CCL11 | -3.1 | NM_002986.2 |
|  | chemokine (C-C motif) ligand 20 | CCL20 | -2.6 | NM_004591.2 |
| *Extracellular Matrix* | | |  |  |
|  | sulfatase 1 | SULF1 | -21.1 | NM_015170.2 |
|  | matrix Gla protein | MGP | -13.1 | NM_000900.3 |
|  | collagen, type I, alpha 2 | COL1A2 | -12.0 | NM_000089.3 |
|  | biglycan | BGN | -6.8 | NM_001711.4 |
|  | collagen, type V, alpha 1 | COL5A1 | -5.4 | NM_000093.3 |
|  | collagen, type IV, alpha 1 | COL4A1 | -4.1 | NM_001845.4 |
|  | matrix metallopeptidase 10 | MMP10 | -4.0 | NM_002425.2 |
|  | integrin, beta 1 | ITGB1 | -3.9 | NM_002211.3 |
|  | collagen, type VIII, alpha 1 | COL8A1 | -3.6 | NM_020351.3 |
|  | collagen, type IV, alpha 2 | COL4A2 | -3.4 | NM_001846.2 |
|  | collagen, type V, alpha 2 | COL5A2 | -3.1 | NM_000393.3 |
|  | fibronectin 1 | FN1 | -2.6 | NM_054034.2 |
|  | laminin, alpha 4 | LAMA4 | -2.4 | NM_002290.3 |
|  | integrin, alpha V | ITGAV | -2.3 | NM_002210.3 |
| *Thrombosis / Vasomotor Function* | | |  |  |
|  | von Willebrand factor | vWF | -5.5 | NM_000552.3 |
|  | angiotensin I converting enzyme 1 | ACE | -5.0 | NM_001178057.1 |
|  | multimerin 1 | MMRN1 | -3.6 | NM_007351.2 |
|  | endothelin 1 | ET-1 | -3.5 | NM_001955.4 |
|  | thrombospondin 1 | THBS1 | -2.6 | NM_003246.2 |
|  | endothelin converting enzyme 1 | ECE-1 | -2.1 | ,NM_001397.2 |
| *Growth Factor Signaling* | | |  |  |
|  | insulin-like growth factor 2 | IGF2 | -10.3 | NM_000612.4 |
|  | bone morphogenetic protein 4 | BMP4 | -6.1 | NM_130851.2 |
|  | latent transforming growth factor beta binding protein 1 | LTBP1 | -5.2 | NM_206943.2 |
|  | platelet-derived growth factor beta polypeptide | PDGF-β | -4.7 | NM_002608.2 |
|  | placental growth factor | PGF | -3.3 | NM_001207012.1 |
|  | cysteine rich transmembrane BMP regulator 1 | CRIM1 | -2.6 | NM_016441.2 |
| *Lipid / Metabolic Pathways* | | |  |  |
|  | peroxisome proliferator-activated receptor alpha | PPARα | -5.0 | NM_005036.4 |
|  | leptin receptor | LEPR | -3.1 | NM_001003679.3 |
|  | retinol binding protein 1 | RBP1 | -2.8 | NM_002899.3 |
| *Junction Proteins* | | |  |  |
|  | gap junction protein, alpha 4, 37kDa | Cx37 | -5.8 | NM_002060.2 |
|  | gap junction protein, alpha 1, 40kDa | Cx40 | -3.3 | NM_005266.5 |

### Table S4. Selected Genes That Are Up-Regulated After Over Expressing COUP-TFII

| **Category** | **Gene Name** | **Gene Symbol** | **Fold Change** | **Accession #** |
| --- | --- | --- | --- | --- |
| *Vascular Development / Arterial-Venous Differentiation* | | |  |  |
|  | lymphatic vessel endothelial hyaluronan receptor 1 | LYVE1 | 6.8 | NM_006691.3 |
|  | nuclear receptor subfamily 2, group F, member 2 | COUP-TFII | 4.6 | NM_021005.3 |
| *Extracellular Matrix* | | |  |  |
|  | versican | VCAN | 9.5 | NM_004385.4 |
|  | syndecan 1 | SDC1 | 8.7 | NM_002997.4 |
|  | laminin, gamma 2 | LAMC2 | 5.9 | NM_018891.2 |
|  | collagen, type IV, alpha 6 | COL4A6 | 5.5 | NM_033641.2 |
|  | ADAM metallopeptidase with thrombospondin type 1 motif, 18 | ADAMTS18 | 4.3 | NM_199355.2 |
|  | matrix metallopeptidase 1 | MMP1 | 3.2 | NM_002421.3 |
|  | collagen, type XVII, alpha 1 | COL17A1 | 2.6 | NM_000494.3 |
|  | ADAM metallopeptidase with thrombospondin type 1 motif, 9 | ADAMTS9 | 2.6 | NM_182920.1 |
| *Lipid / Metabolic Pathways* | | |  |  |
|  | lipase, endothelial | LIPG | 3.9 | NM_006033.2 |
| *Thrombosis / Vasomotor Function* | | |  |  |
|  | serpin peptidase inhibitor, clade D (heparin cofactor), member 1 | SERPIND1 Heparin cofactor II | 3.7 | NM_000185.3 |
|  | serpin peptidase inhibitor, clade B (ovalbumin), member 8 | SERPINB8 | 2.4 | NM_198833.1 |
|  | plasminogen activator, tissue | tPA | 2.2 | NM_033011.2 |
|  | tissue factor pathway inhibitor 2 | TFPI2 | 2.1 | NM_006528.2 |

### Table S5. Selected Genes With Higher Expression In Aortic ECs Than Vena Cava ECs

| **Category** | **Gene Name** | **Gene Symbol** | **Fold Change**  **(Aorta *vs*. Vena Cava)** | **Accession #** |
| --- | --- | --- | --- | --- |
| *Vascular Development / Arterial-Venous Differentiation* | | |  |  |
|  | slit homolog 1 | Slit1 | 67.6 | NM_015748 |
|  | Eph receptor B6 (Ephb6), transcript variant 1 | EphB6 | 65.5 | NM_001146351 |
|  | SRY-box containing gene 13 | Sox13 | 28.1 | NM_011439 |
|  | SRY-box containing gene 17 | Sox17 | 13.3 | NM_011441 |
|  | Ephrin A5 (Efna5), transcript variant 1 | Ephrin-A5 | 10.6 | NM_207654 |
|  | Semaphoring 3G | Sema3g | 9.4 | ENSMUST00000090180 |
|  | kinase insert domain protein receptor | Kdr | 8.0 | NM_010612 |
|  | Semaphoring 6C | Sema6c | 7.8 | NM_011351 |
|  | T-box 1 | Tbx1 | 5.3 | NM_011532 |
|  | smoothened homolog | Smo | 4.7 | NM_176996 |
|  | plexin D1 | Plxnd1 | 4.6 | NM_026376 |
| *Cardiovascular Hormone* | |  |  |  |
|  | adrenergic receptor, alpha 2a | Adra2a | 205 | NM_007417 |
|  | androgen binding protein delta | Abpd | 148.3 | NM_001009952 |
|  | adrenergic receptor, alpha 1b | Adra1b | 6.6 | NM_007416 |
| *Growth Factors Signaling* | | |  |  |
|  | platelet derived growth factor, beta | Pdgfb | 4.6 | NM_011057 |
|  | bone morphogenetic protein 4 | Bmp4 | 4.0 | NM_007554 |
|  | platelet derived growth factor, alpha | Pdgfa | 3.5 | NM_008808 |
| *Extracellular Matrix Biosynthesis* | | |  |  |
|  | fibulin 2 | Fbln2 | 13.1 | NM_007992 |
|  | distal-less homeobox 5 | Dlx5 | 10.1 | NM_010056 |
|  | fibulin 5 | Fbln5 | 5.7 | NM_011812 |
|  | integrin alpha M, transcript variant 1 | Itgam | 2.6 | NM_001082960 |
|  | multimerin 2 | Mmrn2 | 2.5 | NM_153127 |
| *Thrombosis / Vasomotor Function* | | |  |  |
|  | thrombospondin | Thbs1 | 8.0 | NM_011580 |
|  | angiotensin I converting enzyme | Ace | 6.4 | NM_009598 |
|  | coagulation factor X | F10 | 2.5 | NM_007972 |
| *Lipid / Metabolic Pathways* | | |  |  |
|  | leptin receptor (Lepr) | Lepr | 8.4 | NM_010704 |
|  | peroxisome proliferator activated receptor alpha | PPARa | 8.0 | NM_011144 |
|  | apolipoprotein C-I | Apoc1 | 3.4 | NM_007469 |
| *Junction Proteins* | | |  |  |
|  | gap junction protein, beta 6 (Gjb6) | Cx30 | 521.4 | NM_001010937 |
|  | gap junction protein, alpha 5 (Gja5) | Cx40 | 189.6 | NM_008121 |
|  | claudin 5 | Cldn5 | 4.7 | NM_013805 |
|  | gap junction protein, alpha 4 (Gja4) | Cx37 | 2.3 | NM_008120 |

### Table S6. Selected Genes With Higher Expression In Vena Cava ECs Than Aortic ECs

| **Category** | **Gene Name** | **Gene Symbol** | **Fold Change**  **(Vena Cava *vs*. Aorta)** | **Accession #** |
| --- | --- | --- | --- | --- |
| *Vascular Development / Arterial-Venous Differentiation* | | |  |  |
|  | wingless-type MMTV integration site 9B | Wnt9b | 91.2 | NM_011719 |
|  | T-box 5 | Tbx5 | 48.1 | NM_011537 |
|  | apelin | Apelin | 27.7 | NM_013912 |
|  | nuclear receptor subfamily 2, group F, member 2 | COUP-TFII | 10.2 | NM_009697 |
|  | endoglin | Eng | 9.4 | NM_001146350 |
|  | activin A receptor, type 1 | Acvr1 | 9.4 | NM_001110204 |
|  | semaphorin 5A | Sema5a | 6.8 | NM_009154 |
|  | lymphatic vessel endothelial hyaluronan receptor 1 | Lyve1 | 6.2 | NM_053247 |
|  | neuropilin 2 | Nrp2 | 5.6 | NM_001077403 |
|  | Eph receptor B4 | Ephb4 | 3.2 | NM_001159571 |
| *Extracellular Matrix Biosynthesis* | | |  |  |
|  | matrix metallopeptidase 23 | Mmp23 | 25.2 | NM_011985 |
|  | laminin, alpha 4 | Lama4 | 15.1 | NM_010681 |
|  | matrix metallopeptidase 2 | Mmp2 | 6.4 | NM_008610 |
|  | laminin, beta 2 | Lamb2 | 6.2 | NM_008483 |
|  | collagen, type IV, alpha 2 | Col4a2 | 5.9 | NM_009932] |
|  | collagen, type XVI, alpha 1 | Col16a1 | 5.8 | NM_028266 |
|  | tissue inhibitor of metalloproteinase 4 | Timp4 | 5.8 | NM_080639 |
|  | collagen, type V, alpha 3 | Col5a3 | 5.3 | NM_016919 |
|  | tissue inhibitor of metalloproteinase 2 | Timp2 | 4.8 | NM_011594 |
|  | collagen, type III, alpha 1 | Col3a1 | 3.7 | NM_009930 |
|  | a disintegrin-like and metallopeptidase (reprolysin type) with thrombospondin type 1 motif, 2 | Adamts2 | 3.7 | NM_175643 |
| *Thrombosis / Vasomotor Function* | | |  |  |
|  | serine (or cysteine) peptidase inhibitor, clade A, member 3N | Serpina3n | 68.2 | NM_009252 |
|  | serine (or cysteine) peptidase inhibitor, clade G, member 1 | Serping1 | 18.2 | NM_009776 |
|  | plasminogen activator, tissue | tPA | 6.3 | NM_008872 |
|  | tissue factor pathway inhibitor, transcript variant 2 | TFPI2 | 5.3 | NM_001177319 |
|  | serine (or cysteine) peptidase inhibitor, clade B, member 6d | Serpinb6d | 3.8 | NM_001076790 |
| *Cardiovascular Hormone* | |  |  |  |
|  | natriuretic peptide type A | Nppa | 221.9 | NM_008725 |
|  | natriuretic peptide type C | Nppc | 38.0 | NM_010933 |
| *Junction Proteins* | | |  |  |
|  | claudin 11 | Cldn11 | 57.3 | NM_008770 |

### Table S7. Selected Differentially Regulated Genes By COUP-TFII Under TGF-β2 Treatment

| **Category** | **Gene Name** | **Gene Symbol** | **Fold Change**  **(COUP-TFII *vs*. Control RNAi)** | **Accession #** |
| --- | --- | --- | --- | --- |
| *Endothelial-to-Mesenchymal Transition* | | |  |  |
|  | calponin 1, basic, smooth muscle | CNN1 | 8.6 | NM_001299.4 |
|  | collagen, type I, alpha 2 | COL1A2 | 5.9 | NM_000089.3 |
|  | CD44 molecule | CD44 | 5.4 | NM_000610.3 |
|  | serpin peptidase inhibitor, clade E (nexin, plasminogen activator inhibitor type 1), member 2 | SERPINE2/PAI-1 | 4.1 | NM_006216.3 |
|  | transgelin | TAGLN/SM22a | 3.9 | NM_003186.3 |
|  | snail homolog 2 | SNAI2 | 2.4 | NM_003068.4 |
| *TGF-β Signaling Pathway* | | |  |  |
|  | transforming growth factor, beta-induced, 68kDa | TGFBI | 23.1 | NM_000358.2 |
|  | follistatin | FST | 10.6 | NM_006350.3 |
|  | inhibin, beta A | INHBA | 7.5 | NM_002192.2 |
|  | transforming growth factor, beta 2 | TGFB2 | 5.5 | NM_003238.3 |
|  | SMAD family member 6 | SMAD6 | 2.9 | NM_005585.4 |

**SI References:**

1. Dai G*, et al.* Distinct endothelial phenotypes evoked by arterial waveforms derived from atherosclerosis-susceptible and -resistant regions of human vasculature. *Proc Natl Acad Sci U S A* **101**, 14871-14876 (2004).

2. Lee TI, Johnstone SE, Young RA. Chromatin immunoprecipitation and microarray-based analysis of protein location. *Nat Protoc* **1**, 729-748 (2006).

3. Iiyama K*, et al.* Patterns of vascular cell adhesion molecule-1 and intercellular adhesion molecule-1 expression in rabbit and mouse atherosclerotic lesions and at sites predisposed to lesion formation. *Circ Res* **85**, 199-207 (1999).

4. Wang C, Qin L, Manes TD, Kirkiles-Smith NC, Tellides G, Pober JS. Rapamycin antagonizes TNF induction of VCAM-1 on endothelial cells by inhibiting mTORC2. *J Exp Med* **211**, 395-404 (2014).

### Supporting Figures


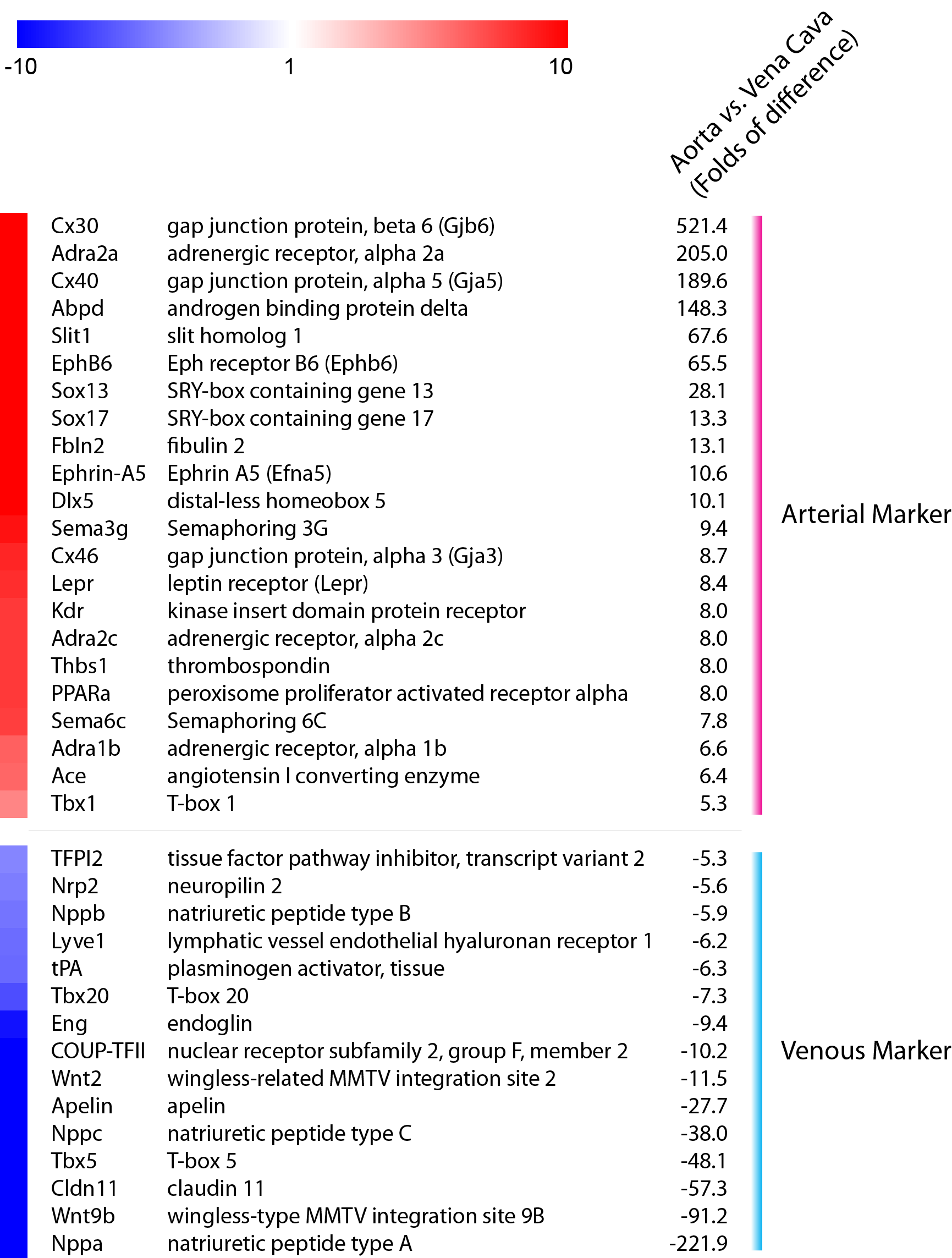


**Figure S1. Arterial and venous ECs demonstrate distinct gene expression pattern.** Some highly differentially expressed genes in aorta or vena cava ECs are listed. All genes listed here are statistically different in aorta *vs*. vena cava.


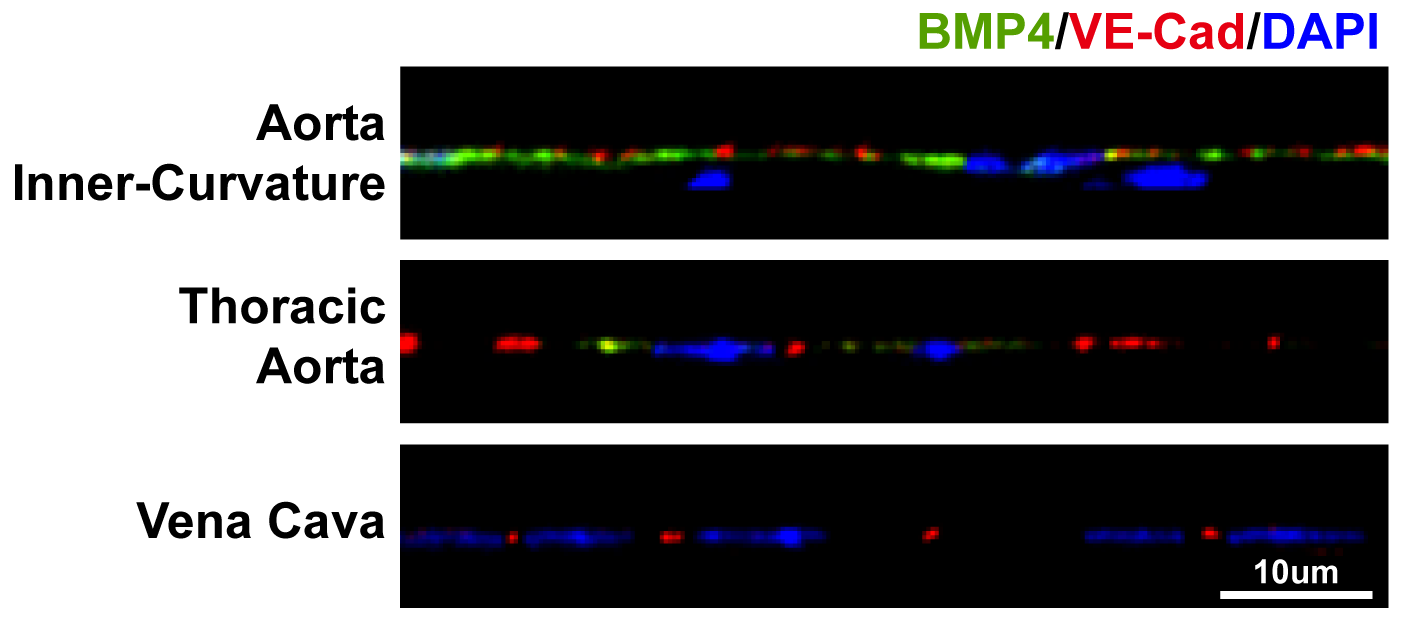


**Figure S2. BMP4 protein expresses only in the endothelium layer of mouse aorta.** Side view (x-z plane) of the *en face* confocal immunofluorescent images of BMP4 expression in mouse aorta and vena cava.


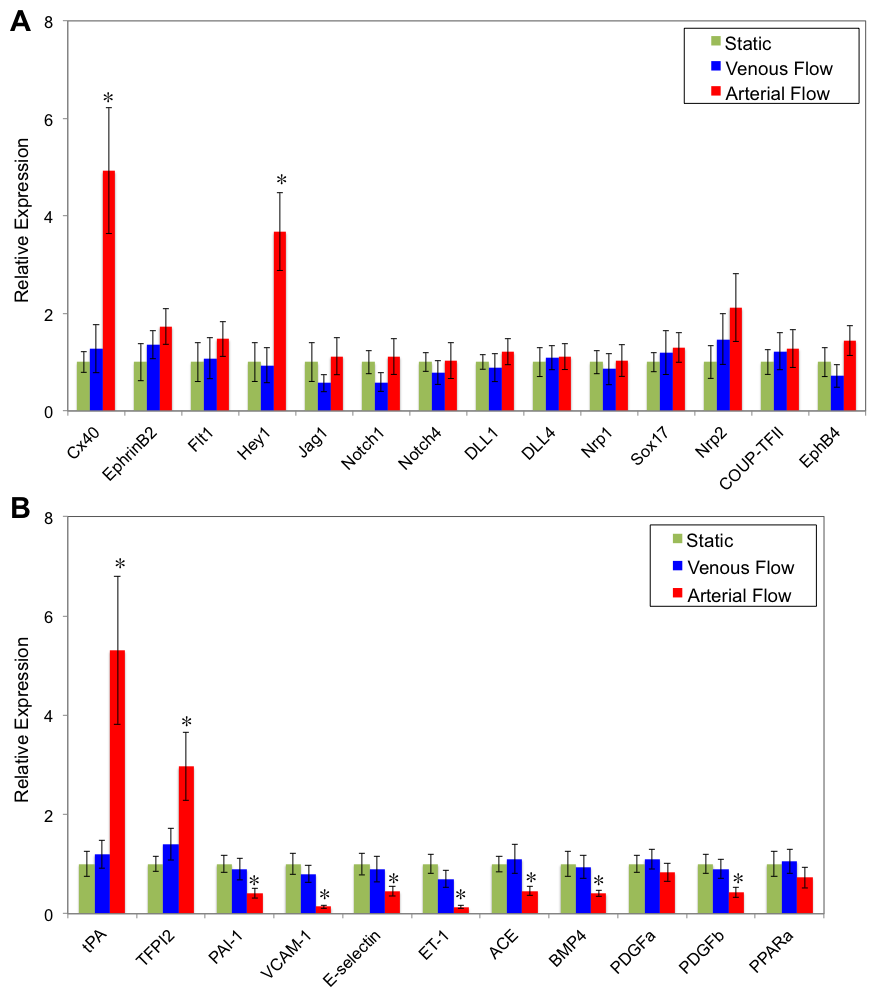


**Figure S3. Arterial flow promotes athero-protective phenotype but has very small influence on arterial venous markers.** HSVECs were cultured under static (no flow), venous or arterial flow condition for 72 hours. Gene expression was analyzed by Taqman RT-PCR, n=3, **p*<0.05.


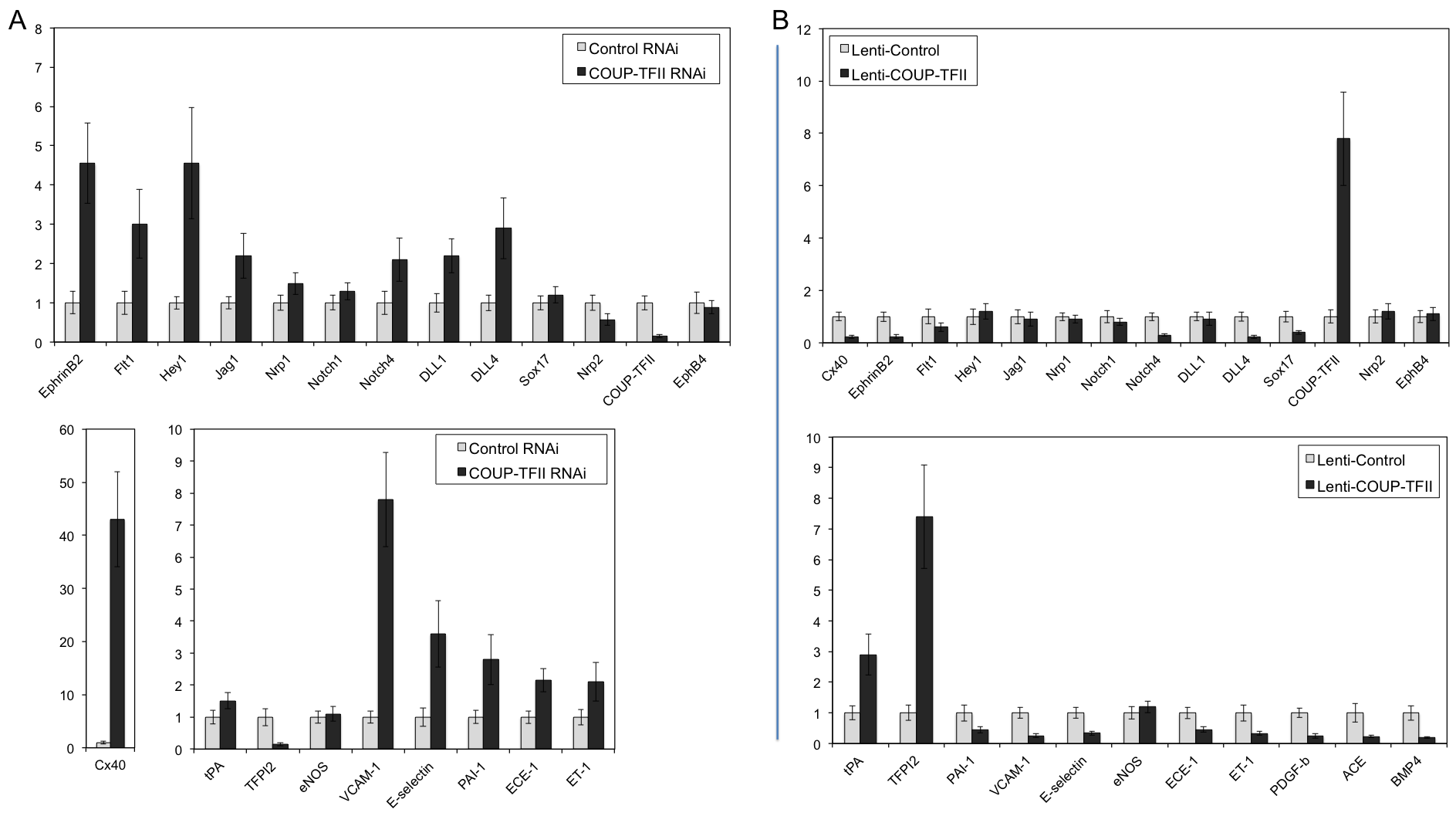


**Figure S4.** (A) HAECs were transfected with COUP-TFII or Control RNAi (10nM). RNA was collected 72 hours after transfection. Gene expressions were measured by Taqman-RT PCR and normalized to RNA 18S. Data are represented as relative expression level, n=3. (B) HAECs were infected with lentivirus-COUP-TFII or control lentivirus (10 MOI), RNAs were isolated after confluent monolayer is reached and all cells were permanently infected with lentivirus. Gene expressions were measured by Taqman-RT PCR and normalized to RNA 18S, n=3.


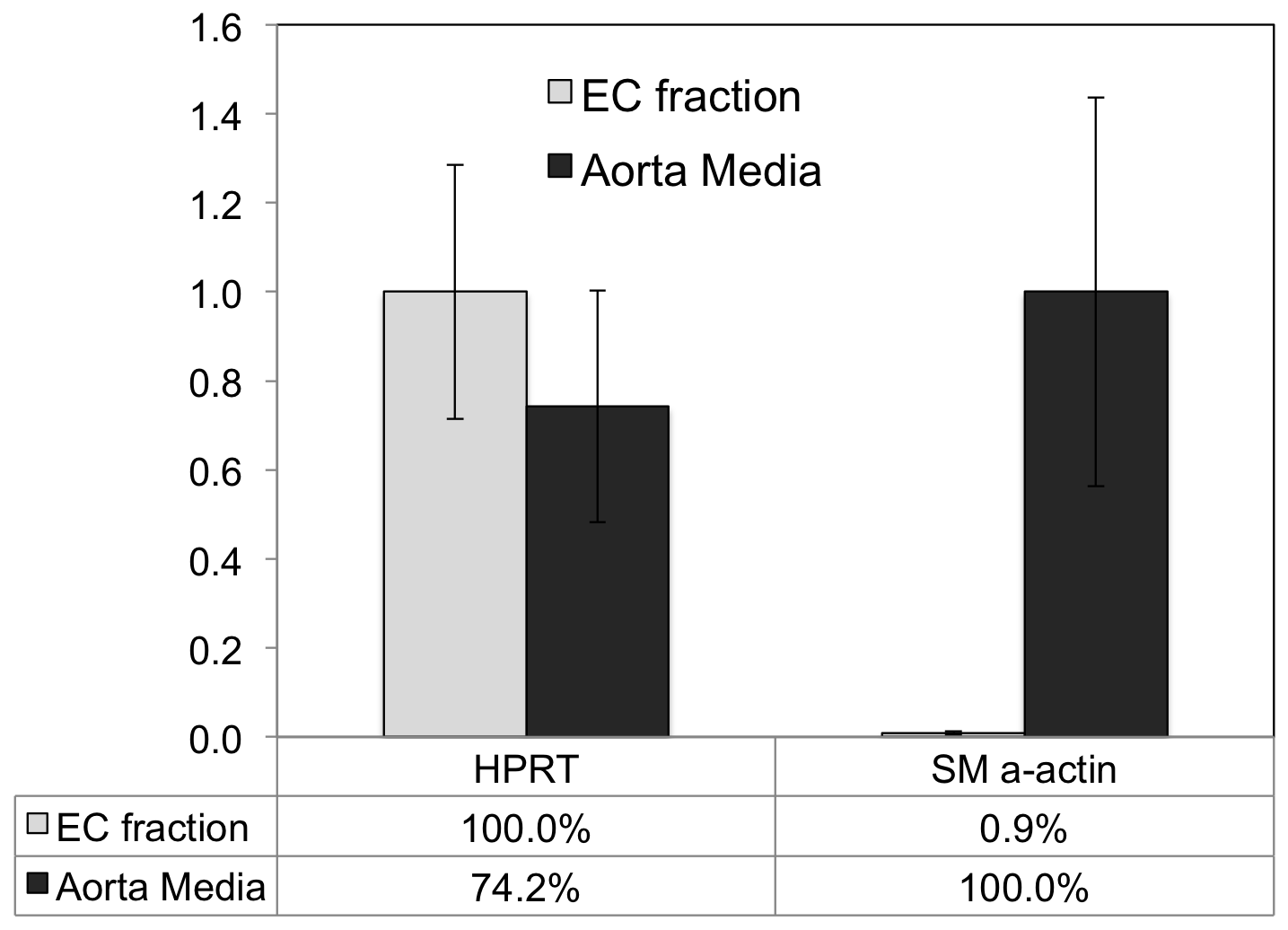


**Figure S5**. RNA was isolated from freshly harvested mouse aorta, and the purity of RNA was checked for smooth muscle contamination by quantifying the expression of smooth muscle alpha actin (SM a-actin) in comparison to RNA samples isolated from aorta media layer. The smooth muscle contamination was determined to be <1%. Hypoxanthine-guanine phosphoribosyltransferase (HPRT) was used as control to verify the equal amount of RNA loading across all samples (n=6).
